# Optical mapping reveals a higher level of large‐scale structural variants in a family with paternally transmitted myotonic dystrophy and independent Parkinson's disease

**DOI:** 10.1002/path.70084

**Published:** 2026-06-09

**Authors:** Md Mehedi Hasan, Jenna Craddock, Tingting Gong, Ruth J Lyons, Igor Stevanovski, Sanjog R Chintalaphani, Ira W Deveson, Weerachai Jaratlerdsiri, Kishore R Kumar, Vanessa M Hayes

**Affiliations:** ^1^ Ancestry and Health Genomics Laboratory, Charles Perkins Centre, School of Medical Sciences, Faculty of Medicine and Health University of Sydney Camperdown New South Wales Australia; ^2^ School of Health Systems and Public Health, University of Pretoria Pretoria South Africa; ^3^ Human Phenome Institute Fudan University Shanghai PR China; ^4^ Cancer Ecosystems Program Garvan Institute of Medical Research and The Kinghorn Cancer Centre Darlinghurst New South Wales Australia; ^5^ Genomics and Inherited Disease Program The Garvan Institute of Medical Research Darlinghurst New South Wales Australia; ^6^ Centre for Population Genomics The Garvan Institute of Medical Research Darlinghurst New South Wales Australia; ^7^ Faculty of Medicine and Health University of New South Wales Sydney New South Wales Australia; ^8^ Computational Genomics Group, Charles Perkins Centre, School of Medical Sciences, Faculty of Medicine and Health University of Sydney Camperdown New South Wales Australia; ^9^ ANZAC Research Institute, Neurology Department and Molecular Medicine Laboratory, Concord Repatriation General Hospital, Sydney Local Health District University of Sydney Concord New South Wales Australia; ^10^ Manchester Cancer Research Centre University of Manchester Manchester UK; ^11^ Norwich Medical School, University of East Anglia Norwich UK

**Keywords:** myotonic dystrophy type 1, optical genome mapping, paternal inheritance, genomic instability, early‐onset Parkinson's disease

## Abstract

Myotonic dystrophy type 1 (DM1) is a clinically challenging multisystem neuromuscular hereditary disorder, with generational increase in severity and earlier age at onset. It is caused by an unstable cytosine‐thymine‐guanine repeat expansion at the *DMPK* locus, accompanied by associated genetic and epigenetic modifications. While somatic mosaicism and meiotic instability are well established, to the best of our knowledge, no study has performed a genome‐wide interrogation for global inherited instability. Performing whole‐genome optical mapping, with sequence base‐resolved structural variant verification, we examine global inherited genomic instability in an atypical paternally transmitted DM1 family presenting with a range of neurological manifestations, including early‐onset Parkinson's disease (PD). While the juvenile‐onset DM1 proband presented with a 10‐fold repeat expansion with associated hypermethylation, her partially hypermethylated asymptomatic protomutation father transmitted a 1.8‐fold contracted allele in the younger premutation sibling. Adult‐onset symptomatic DM1 and PD phenotypic paternal aunts showed significant genome‐wide copy number alteration, including PD‐associated chr19 aneuploidy loss, with additional losses on chr16, 17, and 22. In the absence of potentially pathogenic *de novo* or maternally inherited structural variants, the proband presented with large paternally inherited aberrations impacting gene candidates *CASC15*, *CBFA2T3*, *GPHN*, *H3F3A*, *SDK1*, and *SPAG16*, with advanced global hypomethylation. Here we suggest that inherited genomic instability may contribute to phenotypic variability, including multi‐neurological presentations and single‐generation repeat expansion or contraction. By providing a landscape of inherited large structural variants, this single‐family study expands knowledge of this broad and growing class of diseases. © 2026 The Author(s). *The Journal of Pathology* published by John Wiley & Sons Ltd on behalf of The Pathological Society of Great Britain and Ireland.

## Introduction

Myotonic dystrophy type 1 (DM1) is an autosomal‐dominant inherited multisystem disorder with a prevalence of 0.5 to 18.1 per 100,000 [1]. Due to abnormal muscle fibre membrane electrical activity, patients experience delayed muscle relaxation after voluntary contraction (myotonia), resulting in progressive weakness of the skeletal and smooth muscle [2]. The clinical presentation of DM1 is highly heterogeneous, with variation in both age at onset and disease severity, ranging from mild late‐adult‐onset forms to severe congenital disease [3]. Congenital DM1 is associated with significant morbidity and mortality but is not universally fatal. In non‐congenital forms, clinical features extend beyond myotonia to include muscle wasting, cataracts, cardiac conduction abnormalities, endocrine dysfunction, and neuropsychiatric manifestations [2, 3]. A defining feature of DM1 is the anticipation phenomenon, whereby disease onset typically occurs earlier and with increased severity in successive generations, contributing to clinical complexity and (on average 7 years) delayed diagnoses [3].

The molecular basis of DM1 is an unstable cytosine‐thymine‐guanine (CTG) trinucleotide repeat expansion in the 3’ untranslated region of the dystrophia myotonica protein kinase (*DMPK*) gene on chr19q13.3 [4]. Premutation and expanded alleles have been shown to be unstable, with repeat length variability observed intergenerationally and somatically [5]. At the RNA level, expanded cytosine‐uracil‐guanine transcripts accumulate in nuclear foci and sequester RNA‐binding proteins, including Muscleblind‐like (MBNL) proteins and CELF1, resulting in widespread disruption of alternative splicing across multiple tissues [6, 7]. Based on repeat length, the CTG repeat has been classified as premutation (36–50 repeats), protomutation (51–80 repeats), small expansions (81–150 repeats), intermediate expansions (151–1,000 repeats), or large expansions (> 1,000 repeats) [3]. Larger CTG repeat sizes are generally associated with earlier disease onset and increased severity, ranging from asymptomatic, adult, and juvenile onset to severe or congenital DM1. Consequently, defining repeat lengths within families not only enables diagnosis for asymptomatic and late‐onset cases but also facilitates family planning and clinical management for impacted members.

Genetic testing for DM1 has traditionally focused on repeat length determination using Southern blotting and repeat‐primed amplification, which are labour‐intensive and limited in resolution. Short‐read next‐generation sequencing (NGS) approaches face additional challenges due to the repetitive, guanine‐cytosine‐rich nature of the *DMPK* locus and the size of large expansions [8]. The presence of repeat interruptions further complicates interpretation, with interrupted expansions often associated with milder or later‐onset disease [4, 9]. Addressing these limitations, co‐authors have established an Oxford Nanopore Technology (ONT)‐based targeted long‐read NGS approach providing much needed CTG repeat resolution [10]. Beyond repeat size alone, clinical severity and age at onset are influenced by somatic mosaicism, tissue‐specific instability, and DNA methylation flanking the repeat region, particularly at CTCF‐binding sites [11, 12]. In parallel, damaging *de novo* and inherited genomic variants acquired during gametogenesis are recognised contributors to neurodevelopmental and neurodegenerative disorders [3, 13]. While congenital DM1 is almost exclusively maternally transmitted [3], *de novo* and inherited genomic variation more broadly shows a prenatal bias, influenced by paternal age at conception [14, 15]. However, the landscape and inheritance of genome‐wide large‐scale structural variants (SVs), including genome‐wide methylation status, within DM1 patients with differing *DMPK* expansions and phenotypic presentation has not been systemically explored.

Here, we investigate a two‐generational paternally transmitted DM1 family with marked intrafamilial phenotypic heterogeneity, from juvenile‐onset to asymptomatic DM1, which includes an unrelated (non‐*DMPK* expansion) case of early‐onset Parkinson's disease (PD). The unusual co‐presentation of PD suggests broader neurological heterogeneity in this family, warranting further exploratory genome‐wide investigation. As neuromuscular conditions are associated with large‐scale genomic events, we sought to use a relatively under‐appreciated non‐sequencing high‐resolution imaging technology, optical genome mapping (OGM) [16, 17, 18], to capture the complete spectrum of inherited and *de novo* genomic complexity for the six representative family members, providing further proband‐parent trio long‐read SV validation and genome‐wide methylation predictions. Describing the complete landscape of large‐scale genomic complexity in this single family, which includes notable neuromuscular‐related impacted genes, calls for further studies to elucidate their contribution (if any) to clinical presentation.

## Materials and methods

### Ethical approval and patient consent

Core family members provided written informed consent to undergo both regionally targeted and untargeted whole‐genome investigation as approved by the St Vincent's Human Research Ethics Committee (2019/ETH12538) and the Garvan Institute of Medical Research Governance, under senior author KRK, with Material Transfer Agreement (MTA) executed with the University of Sydney under senior author VMH. All contributing family members provided written consent for publication.

### Study participants

The proband (Lab18) and core family members, mother (Lab17), father (Lab20), brother (Lab19), and paternal aunts with a PD phenotype (Lab21) and DM1 phenotype (Lab22), provided written informed consent to undergo both regionally targeted (diagnostic) and untargeted whole‐genome (exploratory) investigation. The study, approved under ethical review, includes genetic counselling and continued review for all family members. The additional DM1‐phenotypic paternal aunt (Lab23) and her mother (Lab24) were unavailable for sampling due to residing overseas. Both provided verbal consent and access to clinical records, including symptom‐driven and Southern blot (> 100 repeats) definitive DM1 diagnosis for the aunt. Transmission was assumed to be paternal, through clinical and genetic exclusion of the mother.

### Targeted DM1 diagnoses

Using our targeted ONT NGS methodology [10], *DMPK* read lengths supporting genetic diagnosis were provided for the proband and core family members. In brief, high‐molecular‐weight (HMW) DNA extracted using PacBio Nanobind CBB kit (Pacific Biosciences, Menlo Park, CA, USA) underwent shearing to ~30‐kb fragments using the Megaruptor 3 system (Diagenode, Liège, Belgium) and visualised on an Agilent Femto Pulse using the Genomic DNA 165 kb Kit (Agilent Technologies, Santa Clara, CA, USA). ONT libraries, prepared from ~3 μg of sheared DNA (ligation prep, SQK‐NBD114.24), were barcoded, pooled (three samples), and loaded on an ONT PromethION R10.4.1 flow cell and sequenced using either a PromethION 2 Solo or PromethION 48 instrument (Kinghorn Cancer Centre, Garvan Institute of Medical Research, Darlinghurst, NSW, Australia). Live target selection/rejection was executed using the Readfish software package [19] for a targeted panel of known pathogenic repeats, including the *DMPK* region [10]. Reads were retrieved within a 50‐kb window, assembled *de novo* using Flye (version 2.8.1‐b1676; https://github.com/fenderglass/Flye, last accessed: 19 June 2024) to create a pseudo‐haploid contig to which reads were realigned, phased into separate haplotypes using Longshot (version 0.4.1; https://github.com/pjedge/longshot, last accessed: 19 June 2024), and re‐polished with reads from each haplotype using Racon (version 1.4.0; https://github.com/isovic/racon, last accessed: 19 June 2024) to generate two distinct haploid contigs. The precise position was identified by mapping 150‐bp unique flanking sequences extracted from T2T‐CHM13 version 2.0 using minimap2 (version 2.22; https://github.com/lh3/minimap2, last accessed: 19 June 2024).

### Optical genome mapping

For the proband and core family members, HMW DNA was isolated from whole blood using the Bionano Genomics Blood and Cell Culture DNA Isolation Kit (Bionano Genomics, San Diego, CA, USA). Agarose matrix immobilisation ensures DNA molecules remain intact (> 150 kb) during cell lysis and purification. Molecules were fluorescently tagged (6–7 base motif) using the Bionano Genomics DLS DNA Labeling Kit (Bionano Genomics), with DLE‐Green fluorophores and DLE‐1 enzyme incubation with proteinase K deactivation. Loaded onto a Saphyr Chip and using the Saphyr instrument (Hayes Lab, University of Sydney, Australia), through electrophoresis, labelled DNA molecules were unwound, linearized, and passed through nanochannels, electrophoresis paused, and molecules imaged. Each image provided a collection of labels with estimated distances (averaging 14–17 labels per 100 kp), creating a merged BNX data file which was automatically *de novo* assembled into a refined consensus and diploid‐resolved genome map using the Bionano Solve software (Bionano Genomics).

### OGM‐identified structural variation

Unlike traditional short‐read NGS technologies, OGM‐derived reference free consensus maps allow for unbiased visualisation (rather than inference) of megabase‐sized SVs, through aligning multiple maps against an GRCh38 *in silico* labelled human reference, utilising the Bionano Genomics (BNG) Access software on Saphyr Compute Servers (https://bionano.com/compute-solutions/, last accessed: 28 January 2026). Against this reference, SVs are detected as differences in distances between labels, loss (deletions) or gain of material (insertions), or label repetition (duplications) or orientation (inverted) or position (inversion or translocation). Polymorphic SVs were filtered out against the BNG control public database (297 healthy individuals). To further pinpoint SVs unique to each individual, a series of filters were applied including (i) minimum SV size of 500 bp, (ii) variant allele frequency (VAF) range from 0 to 1, (iii) occurrence rate of ≤ 100% in control samples and ≤ 0% in the broader BNG control database, (iv) self‐molecule count of ≥ 5, (v) exclusion of samples with failing chimeric scores or overlapping genes, (vi) minimum copy number variant (CNV) size of 50 kb, and (vii) minimum size for absence of heterozygosity/loss of heterozygosity (AOH/LOH) of 0 bp. The CNV pipeline identified large, unbalanced aberrations through normalised molecule coverage, whereas the SV pipeline detected variants by comparing labelling patterns between the sample genome map and reference. To ensure detection of inherited over acquired variants, confidence was set to 0.05, with further confirmation of VAFs.

### OGM‐derived structural variant validation and methylation prediction

Both long‐ and short‐read untargeted NGS technologies were used for OGM‐derived SV validations. In brief, HMW DNA extracted from the proband trio underwent shallow whole‐genome long‐read HiFi NGS using the PacBio Sequel II system (Australian Genome Research Facility, Melbourne, VIC, Australia) generating coverages of 11.43× (proband), 10.91× (mother), and 13.04× (father). Consensus reads were analysed and aligned to GRCh38 using the SMRTlink 8.0 software allowing for OGM‐derived potentially pathogenic SVs to be validated through read‐specific visualisation using the Integrative Genomics Viewer (IGV). Additionally, PacBio data allowed for methylation probability analysis, conducted utilising pb‐CpG‐tools version 2.3.1 (https://github.com/PacificBiosciences/pb-CpG-tools, last accessed: 30 November 2023) with default parameters. Further SV breakpoint validation for the proband was derived from 2 × 150 cycle paired‐end short‐read whole‐genome sequencing using the Illumina NovoSeq 6000 system (University of New South Wales Ramaciotti Centre for Genomics, Randwick, NSW, Australia). Generating 39.44× coverage, short reads were aligned to GRCh38 with alternative contigs using scalable FASTQ‐to‐BAM (version 2.0; https://github.com/PacificBiosciences/pbbioconda, last accessed: 28 January 2026) workflow with default settings and adhering to Broad Institute's best practice recommendations [20]. Reads spanning candidate pathogenic OGM‐derived SVs were further visualised using manual inspection through IGV.

## Results

### Proband clinical presentation

**Figure 1:**
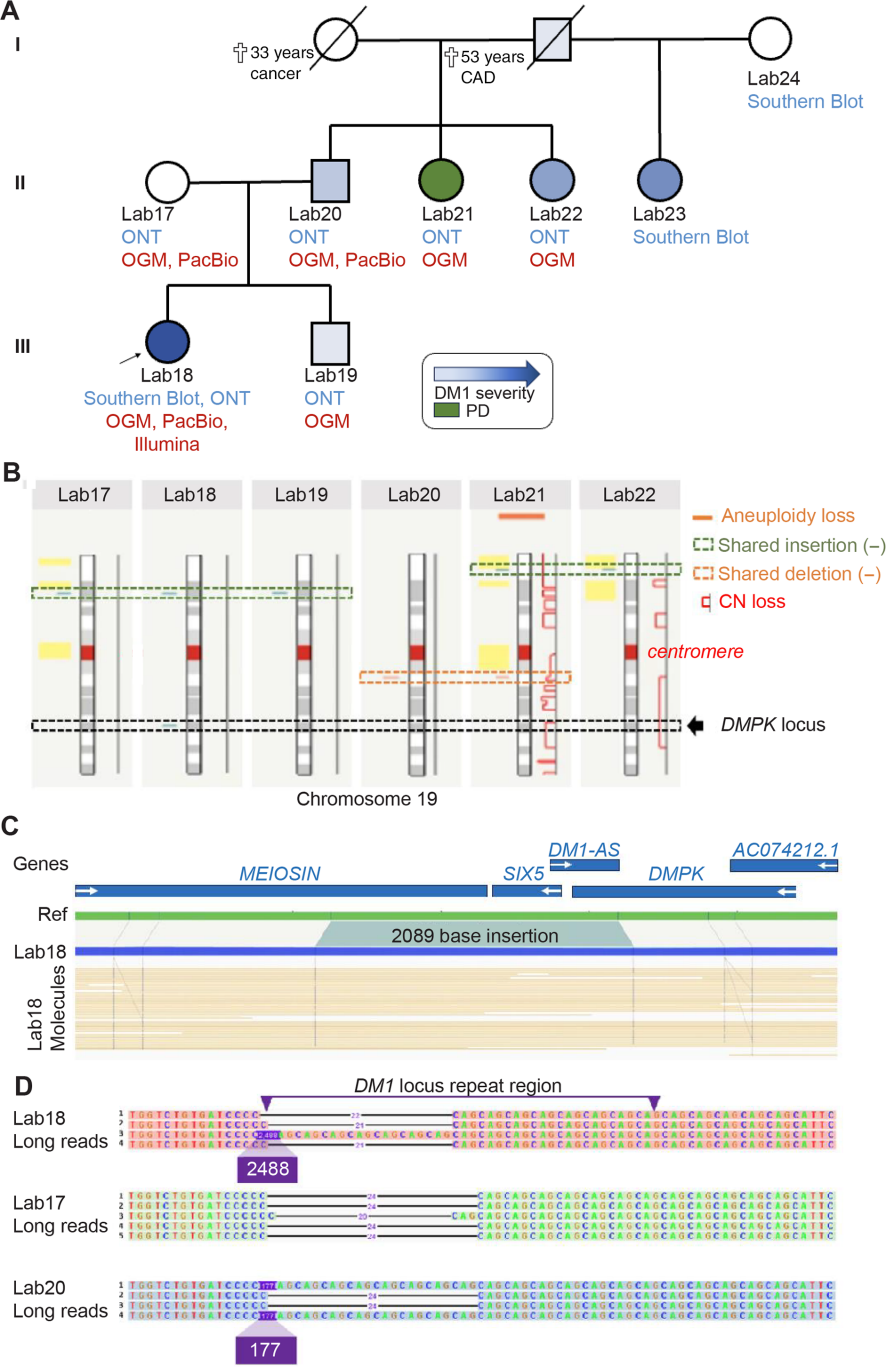
DM1 family with chr19 DM1 locus optical mapping and long‐read trio‐based repeat resolution. (A) Three‐generation family tree for proband (Lab18) and extended paternal DM1 (blue shade showing DM1 severity) family members, including premutation sibling (Lab19), protomutation father (Lab20), DM1 symptomatic paternal aunts (Lab22 and Lab23), and PD (green) phenotypic paternal aunt (Lab21). It can be assumed that transmission was via the paternal grandfather. Both the proband's mother (Lab17) and paternal half‐aunt's mother (Lab24) were genetically confirmed as non‐carriers. (B) Optical genome mapped chr19‐derived cytobands for extended family depicting location of unique, including shared, large insertion (green), and deletion (orange) events, a large *DMPK*‐associated insertion at the DM1 locus in the proband (Lab18), copy number (CN) loss in Lab21 and Lab22 (paternal aunts), including chromosomal aneuploidy loss (orange) for Lab21 (PD phenotypic paternal aunt), and regions (yellow) of AOH or LOH. (C) Focusing in on the DM1 locus for Lab18 provides deep OGM molecule support for a clinically relevant roughly 2,089 base insertion event disrupting *DMPK* and neighbouring genes. (D) PacBio long‐read validation establishing DM1 loci repeat length estimations for the proband (Lab18, 13/849 alleles), her unaffected mother (Lab17, 12/13 alleles), and transmitting father (Lab20, 12/79 alleles).

The female proband (Lab18, Figure 1A) was born prematurely (29 weeks' gestation) with no clinical manifestations of DM1. She had pneumothorax within 24 h of birth requiring treatment with supplemental oxygen. Concerns regarding dysmorphic facies (large facial features) prompted karyotyping, which revealed no chromosomal abnormalities. Milestones were delayed, with gastroesophageal reflux and difficulty maintaining nutrition during the first 2 years of life, with no medical cause identified. She was diagnosed with severe anxiety disorder (at age 3 years), which included social, generalized, and separation anxiety disorder, with specific phobias. The frequency and severity of panic attacks increased steadily with age (treated with agomelatine). The proband reported awareness of muscular weakness of the arms and legs beginning around 8 to 10 years of age, with an autism level 1 diagnosis in her mid‐teens. Early medical observations with no definitive explanations included lack of thriving, excessive sleeping or day‐time exhaustion, and nocturnal enuresis. The proband was hospitalised on multiple occasions with inconclusive findings. She had recurrent urinary tract infections attributed to urinary retention. Late teens saw DM1‐like symptoms becoming exacerbated including myotonia and muscular weakness in hands and ankles, worsening of pigeon‐toed (intoeing) appearance, drooping of the head, skewed smile and ptosis, tachycardia, scalp psoriasis, reduced capacity to swallow and chew, and severe sleep apnoea. The proband experienced hormonally induced pulmonary embolism (20 years), DM1 diagnosis (21 years, Southern blot > 700 repeats), and surgical treatment for endometriosis (23 years).

### Complex family history, genetic diagnosis, and transmission

With no prior history of DM1 in either family, the maternal grandparents lived well into their 80s, but the paternal grandmother died at 33 years from cervical cancer and grandfather at 53 years from myocardial infarction (Figure 1A). The myocardial infarction was attributed, through extensive genetic testing of the father's large (11 siblings) family, to a genetically confirmed and cholesterol level‐associated pathogenic‐verified variant in the *LDLR* gene (c.1329G>C, p.Trp443Cyc), consistent with familial hypercholesterolaemia (FH) [21]. However, this interpretation was made in the context of recognised cardiac involvement in DM1. The proband, father, and brother inherited FH, with the proband using PCK9 inhibitors to maintain cholesterol levels. The first indication of neurological and/or neuromuscular degenerative conditions in the family included early‐onset (45 years) PD diagnosis for the paternal aunt (Lab21). Given the lack of DM1‐associated phenotypic features, PD was assumed to be an independent manifestation. The first indication of DM1 came from the paternal half‐aunt (Lab23), manifesting as muscle weakness followed by genetic confirmation at 28 years of age. Paternal transmission was assumed through maternal exclusion (Lab24). With no obvious or debilitating features at 50 years, the father (Lab20) had pronounced frontal balding, the most common male‐associated trait for mild DM1 presentation [3]. The DM1‐phenotypic aunt (Lab22) in her early 40s had all symptoms associated with classical DM1, including myotonia and falling, facial weakness and voice alterations, and general fatigue. The proband's brother (Lab19) and mother (Lab17) showed no clinical features during neurological testing. Using our targeted ONT diagnostic approach [10] (supplementary material, Figure S1), uninterrupted CTG repeat lengths were estimated for the proband (13/777 repeats), brother (11/47), mother (11/13), father (12/83), paternal PD aunt (12/16), and paternal DM1 aunt (5/178). This confirmed paternal transmission from a protomutation or small expansion carrier, leading to exacerbated repeat expansion in the proband and contraction in the premutation younger sibling. Except for the PD aunt, all paternal siblings inherited varying degrees of paternally transmitted repeat lengths and DM1 presentation. Here, we aimed to determine the complete spectrum of shared and unique paternally over maternally inherited large‐scale SVs in the proband and her impacted family members, which would provide a window into the extent of genomic complexity within this single unusual DM1 family, including assumed DM1‐independent PD manifestation.

### DM1 locus exploration using OGM with long‐read base estimates

Using OGM we generated effective average coverages of 83.54× (mother), 83.68× (proband), 170.29× (brother), 224.26× (father), 76.13× (PD‐aunt), and 77.11× (DM1‐aunt) for the family (supplementary material, Table S1). Targeting fragmental rearrangements over 500 bases (lower threshold), OGM confirmed clinically significant chr19 DM1 locus expansion in the proband (Figure 1B; supplementary material, Figure S2). Outside the DM1 locus, large‐scale chr19 SVs shared among family members included a maternally inherited ~2.2‐kb non‐gene‐impacting insertion shared by the offspring, an almost 5‐kb *TMIGD2* impacting insertion shared between the paternal aunts, and a roughly 5.2‐kb *TDRD12* impacting deletion shared between father and PD‐aunt. Notably, both paternal aunts show substantial chr19 copy number (CN) loss, including aneuploidy in the PD‐aunt. Genome maps spanning the DM1 locus revealed in the proband an estimated 2,089‐base heterozygous insertion upstream of *DMPK* and including neighbouring genes *SIX5*, *MEIOSIN*, *DM1‐AS*, and *AC074212.1* (Figure 1C). As OGM is not base specific, shallow trio‐based PacBio sequencing provided repeat length estimates of 13/849 (three versus one read) for the proband, 12/79 (two reads each) for the father, and 12/13 (one versus four reads) for the mother (Figure 1D), with further target‐specific short‐read breakend validation for the proband (supplementary material, Figure S3). While our study highlights the variability in repeat lengths by technology (diagnostic versus explorative), together they provide a best‐fit genetic diagnosis (Table 1).

**Table 1:** *DMPK* repeat estimations in bases for proband (Lab18) and her family members, using both targeted (diagnostic) and untargeted (exploratory) technologies, with associated clinical DM1 diagnoses.

Family position	Lab17	Lab18	Lab19	Lab20	Lab21	Lab22
	Mother	Proband	Brother	Father	PD Aunt	DM1 Aunt
Targeted genetic testing						
Southern blot	NA	> 700	NA	NA	NA	NA
ONT Allele 1	11	13	11	12	12	5
ONT Allele 2	13	777	47	83	16	178
OGM detection and OGM‐directed Illumina breakpoint (BND) validation						
Map	< 166	> 697	< 166	< 166	< 166	< 166
BND	NA	Validated	NA	NA	NA	NA
PacBio detection*						
Read 1	12	13	NA	79	NA	NA
Read 2	12	13	NA	12	NA	NA
Read 3	13	849	NA	12	NA	NA
Read 4	12	13	NA	79	NA	NA
Read 5	12	NA	NA	NA	NA	NA
DM1 clinical correlation						
	Unaffected	DM1 expansion mutation, classical to severe (juvenile)	DM1 permutation, unaffected	DM1 protomutation, asymptomatic to mild	Unaffected	DM1 expansion mutation, classical (adult)

* While five PacBio reads were generated for Lab17, both Lab18 and Lab20 presented with four reads each over the *DMPK* locus.

BND, breakpoint; DM1, myotonic dystrophy type 1; NA, non‐applicable; ONT, Oxford Nanopore Technology; PD, Parkinson's disease.

### OGM‐derived global structural variants with long‐ and short‐read validation

After filtering for commonly observed population‐wide OGM SVs, family members presented with 17 (mother; not shown), 26 (proband; Table 2), 23 (brother; supplementary material, Table S2), 30 (father; supplementary material, Table S3), 23 (PD‐aunt, Table 3), and 33 (DM1‐aunt; supplementary material, Table S4) unique OGM‐derived SVs (supplementary material, Figure S4). Irrespective of DM1/PD status, deletions predominated (96/153, 62.7%) over insertions (47/153, 30.7%), while large duplications are rare (supplementary material, Figure S5). Size estimations ranged from 1 kb to 1.8 Mb. The proband shared proportionately more unique overlapping SVs with her transmitting father 61.53% (16/26) than her premutation sibling 56.52% (13/23), with six shared between the siblings. Furthermore, the proband shared 50% (8/16) of her paternally inherited unique SVs with her DM1‐aunt, compared to 12.5% (2/16) with her PD‐aunt. Neither of the two *de novo* SVs directly impacted a gene of known clinical relevance. In turn, three of eight maternally inherited SVs (VAF range: 0.32–0.59) impacted gene regions of which *SEC23B* and *SLC43A2* have been associated with congenital dyserythropoietic anaemia or Cowden syndrome [22] and alopecia (balding) with palmoplantar keratoderma (thickened soles and palms) [23] respectively, with no links to neuromuscular degenerative diseases. However, within the >230 kb *DOCK8–KANK1* inclusive chr9p24.3 duplication (VAF = 0.47; supplementary material, Figure S6A,B), including both PacBio and Illumina read‐specific breakend validation (supplementary material, Figure S6C), whereas *DOCK8* has been associated with infectious diseases [24], *KANK1* has been associated with neurodevelopmental disorders, including autism [25]. Although duplications (gains) in this region have been reported, loss of function through deletion or translocation, also known as chr9p deletion syndromes, are well established [26]. Intriguingly, when dropping the confidence threshold to −1 to reveal non‐inherited or acquired SVs (VAFs < 0.3), though not the focus of this study, we found the proband to present with a translocation resulting in somatic mosaic 9p24.3 depletion (VAF = 0.17 and maternally absent) originating upstream of *DOCK8–KANK1* with the partner breakpoint on the alternative strand (supplementary material, Figure S6A,B). Notably, neurodevelopmental disorders are the most reported feature for this chromosomal loss.

**Table 2:** Excluding for the DM1 locus, unique global optical genome mapping (OGM) large‐scale structural variants (SVs) identified in the proband (Lab18) by inheritance status (*n* = 26).

Chromosome: position	SV type	Size (bp)	VAF	Overlapping genes*	Nearest non‐overlapping locus^†^	Co‐inherited	PacBio^‡^ or Illumina^§^ validation
Private SVs (*n* = 2)							
chr5: 25952515–25968967	DEL	1,095	0.41	*AC113370.1*	*MSNP1*	NA	ND
chr11:37762260–37818524	DUP (inv)	56,265	0.43	‐	*RPL7AP56*	NA	ND
Paternally inherited SVs (*n* = 16)							
chr1: 113243421–113260985	DEL	3,914	0.49	‐	*AL357055.2*	‐	ND
chr1: 226071312–226074381	INS	6,872	0.59	***H3F3A***	*LINC01703*	‐	PacBio Illumina
chr2: 187671167–187685624	DEL	12,750	0.51	‐	*LINC01090*	Brother	ND
chr2: 213504200–213513769	INS	3,376	0.49	***SPAG16***	*SPAG16‐DT*	Brother DM1‐aunt	PacBio Illumina
chr2: 213566756–213578189	DEL	6,088	0.5	***SPAG16***	*MIR4438*	Brother DM1‐aunt	PacBio Illumina
chr5: 25952515–25968967	DEL	1,095	0.41	*AC113370.1*	*MSNP1*	‐	ND
chr6: 21888982–21899973	DEL	4,799	0.54	***CASC15***	*AL136313.1*	PD‐aunt DM1‐aunt	PacBio
chr7: 3558405–3576211	DEL	9,024	0.5	***SDK1***	*AC011284.1*	PD‐aunt DM1‐aunt	PacBio
chr11: 57957431–57967001	DEL	1,176	0.53	‐	*OR5BD1P*	DM1‐aunt	ND
chr11:37762260–37818524	DUP (inv)	56,265	0.43	‐	*RPL7AP56*	DM1‐aunt	ND
chr11:37738303–37762260	INS	77,319	0.44	‐	*RPL7AP56*	DM1‐aunt	ND
chr12: 54304003–54321540	DEL	10,421	0.57	*AC078778.1* *RNU6‐950P*	*NFE2*	DM1‐aunt	ND
chr14: 46540452–46556096	DEL	4,373	0.41	‐	*LINC00871*	DM1‐aunt	ND
chr14: 66944487–66959894	INS	1,950	0.49	***GPHN***	*AL049835.1*	Brother	PacBio Illumina
chr16: 8092039–8340701	DEL	211,893	0.52	*AC093515.1* *AC018767.2* *LINC02152* *AC018767.3*	*AC018767.1*	Brother	ND
chr16: 88868384–88914625	DEL	5,966	0.25	***CBFA2T3** AC092384.2*	*PABPN1L*	Brother	PacBio
Maternally inherited SVs (*n* = 8)							
chr3: 24848468–24867812	DEL	16,629	0.47	*AC092422.1*	*RN7SL216P*	Brother	ND
chr6: 26727983–26750048	DEL	7,996	0.54	‐	*AL513548.1*	Brother	ND
chr8: 34483569–34492118	DEL	1,632	0.59	‐	*AC090993.1*	Brother	ND
chr9: 296371–534652	DUP	238,282	0.47	*DOCK8* *AL161725.1* ***KANK1*** *RPL12P25* *AL161725.2*	*AL392089.1*	‐	PacBio Illumina
chr19: 10341841–10346616	INS	2,194	0.51	‐	*ICAM3*	Brother	ND
chr20: 18517361–18523874	INS	4,566	0.53	*SEC23B*	*RPS19P1*	‐	ND
chrX: 30594114–30623441	DEL	2,912	0.58	*CKS1BP6*	*FTLP2*	‐	ND
chr17: 1571070–1572385	INS	15,742	0.32	*SLC43A2*	*PITPNA*	‐	ND

* OGM‐derived SVs impacting neuromuscular associated gene candidates are defined in bold.

^†^ Not all entries represent official HUGO gene symbols. Some loci correspond to ENSEMBL‐predicted transcripts or gene models that require experimental confirmation.

^‡^ Manual inspection validation for presence of SV using shallow coverage PacBio long‐reads for Lab18, with data generated for Lab17 and Lab20 providing further validation for maternal versus paternal inheritance.

^§^ Manual inspection validation for SVs using deep Illumina short‐read WGS data for Lab18.

bp, base pairs; chr, chromosome; DEL, deletion; DUP, duplication; INS, insertion; INV, inversion; NA, not applicable; ND, not determined; SV, structural variant; VAF, variant allele frequency; PacBio, long‐read sequencing; Illumina, short‐read sequencing.

**Table 3:** Unique optical genome mapping (OGM)‐derived structural variants (SVs, *n* = 23) identified in paternal aunt (Lab21) with early‐onset Parkinson's disease (PD).

Chromosome: position	SV type	Size (bp)	VAF	Overlapping genes*	Nearest non‐overlapping locus^†^	Co‐inherited
Private SVs (*n* = 6)						
chr1:98542108–98561867	DEL	5,923	0.46	‐	*AC095031.1*	NA
chr10:19218471–19234557	INS	2,498	0.63	***MALRD1***	*AL590378.1*	NA
chr11:31248953–31276580	DEL	2,623	0.49	*DCDC1*	*CYCSP25*	NA
chr11:57872888–57886196	DEL	8,148	0.5	*OR5BA1P*	*AP003484.1*	NA
chr11:97634853–97675313	DEL	38,864	0.56	*RNA5SP347*	*LINC02713*	NA
chr17:39445724–39454220	INS	3,008	0.51	*MED1*	*CDK12*	NA
Shared SVs (*n* = 17)						
chr6:5963134–5967214	DEL	1,055	0.43	‐	*PKMP5*	Father
chr6:21889020–21899935	DEL	4,679	0.42	***CASC15***	*AL136313.1*	Proband, father, DM1‐aunt
chr7:3558427–3576187	DEL	8,994	0.45	***SDK1***	*AC011284.1*	Proband, father, DM1‐aunt
chr8:24843835–24856561	DEL	2,879	0.53	‐	*AF106564.1*	DM1‐aunt
chr8:93158852–93166540	DEL	4,599	0.48	*C8orf87*	*LINC00535*	Brother, father, DM1‐aunt
chr8:93960548–93965700	DEL	1,002	0.62	‐	*RPL34P18*	Brother, father, DM1‐aunt
chr8:99845541–99859871	INS	6,156	0.49	*VPS13B*	*COX6C*	Brother, father, DM1‐aunt
chr8:123946595–123956695	DEL	2,987	0.49	***FER1L6***	*AC090753.1*	Father, DM1‐aunt
chr8:124724677–124749262	DEL	1,319	0.54	*MTSS1*	*AC100858.3*	Father, DM1‐aunt
chr9:86985018–86995213	INS	6,057	0.57	*GAS1RR*	*AL513318.2*	Brother, father
chr10:2271522–2279751	INS	6,406	0.52	‐	*LINC00701*	DM1‐aunt
chr16:68185354–68204527	DEL	1,486	0.48	*NFATC3* *AC130462.1* *AC020978.6* *AC020978.8*	*RPS12P27*	DM1‐aunt
chr16:72321271–72350532	DEL	15,637	0.6	*LINC01572*	*U6*	DM1‐aunt
chr19:4292917–4297930	INS	4,897	0.6	*TMIGD2*	*SHD*	DM1‐aunt
chr19:32733750–32743327	DEL	5,260	0.56	*TDRD12*	*AC008736.2*	Father
chr21:32251481–32266664	DEL	5,374	0.55	*AP000265.1*	*MIS18A*	Brother, father, DM1‐aunt
chrX:41053993–41066578	INS	3,222	0.47	‐	*CLIC4P3*	DM1‐aunt

* OGM‐derived SVs impacting neuromuscular‐associated gene candidates are defined in bold.

^†^ Not all entries represent official HUGO gene symbols. Some loci correspond to ENSEMBL‐predicted transcripts or gene models that require experimental confirmation.

bp, base pairs; chr, chromosome; DEL, deletion; DM1, myotonic dystrophy type 1; INS, insertion; INV, inversion; NA, not applicable; SV, structural variant; VAF, variant allele frequency.

**Figure 2:**
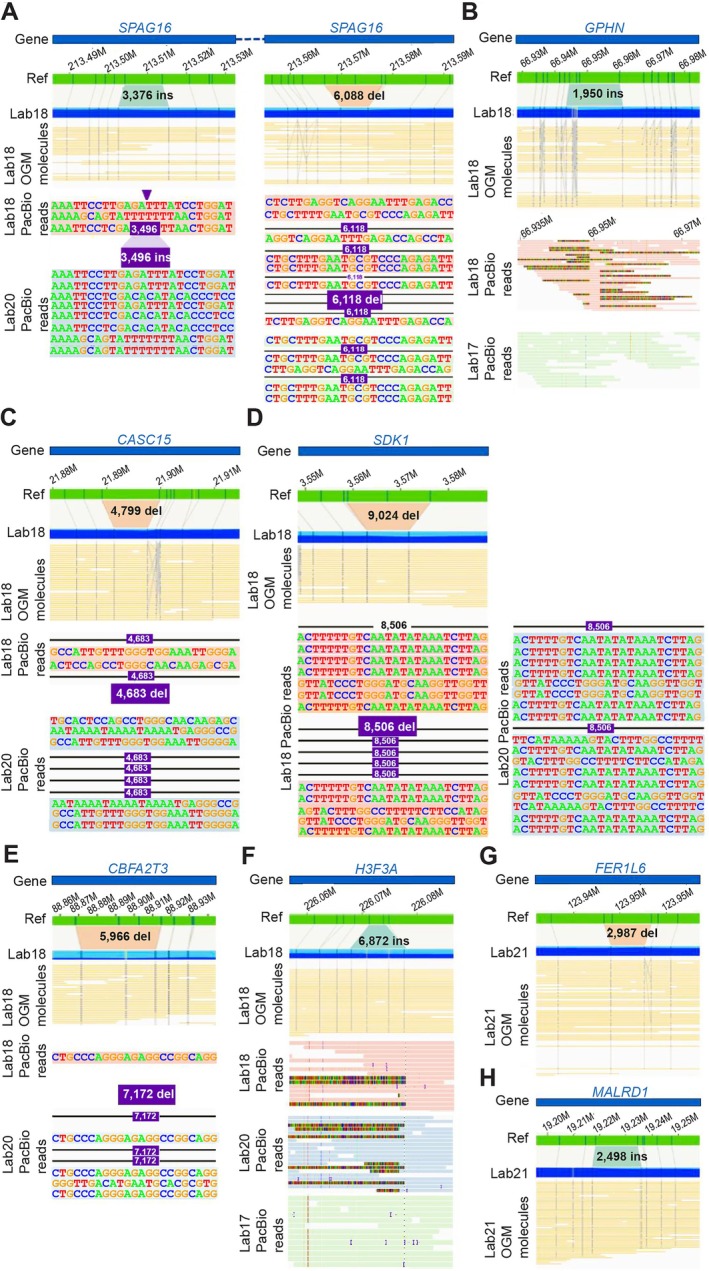
Paternally inherited structural variants identified by OGM and long‐read validation. Clinically relevant paternally inherited OGM‐identified large SVs in proband (Lab18), including size‐estimated insertions (green) and deletions (orange), with sequence base verification using PacBio long‐read manual interrogation for proband (red), her father Lab20 (blue), and/or mother Lab17 (green) for (A) *SPAG16* insertion and linked deletion, (B) *GPHN* insertion, (C) *CASC15* deletion, (D) *SDK1* deletion, (E) *CBFA2T3* deletion, and (F) *H3F3A* insertion. Clinically relevant OGM SVs detected in early‐onset PD phenotypic Lab21 and including (G) *FER1L6* deletion and (H) *MALRD1* insertion.

Of the 16 paternally inherited SVs in the proband, seven SVs impacted six genes of notable clinical relevance, all of which (100%) were validated through PacBio long‐read inspection and four (57%) using Illumina short‐read breakpoint inspection. Potential pathogenic paternally inherited OGM SVs included an approximately 3.4‐kb (PacBio 3,496 bp) insertion and ~6‐kb (PacBio 6,118 bp) deletion (exon 10 overlap) of *SPAG16* (Figure 2A), and an ~2‐kb *GPHN* insertion (exon 8), though not called using PacBio long‐read disruption, was visualised when compared to the mother's intact long reads (Figure 2B), with further short‐read breakend validation (supplementary material, Figure S7A,B). Dysfunction and/or mutation of *SPAG16* and *GPHN* were previously associated with neurodevelopmental and psychiatric disorders, such as autism, intellectual disability, and epilepsy [27], with additional links to schizophrenia and Alzheimer's disease for *GPHN* [28]. While not directly associated with neurodegenerative conditions, the ~4.8‐kb *CASC15* intronic (Figure 2C) and ~9‐kb *SDK1* exon 2 deletions (Figure 2D), validated as 4,683 and 8,506 bp deletions using PacBio manual read inspection, respectively, raised further interest in our study. *CASC15* encodes for a long non‐coding RNA (lncRNA) associated with basal cell carcinoma and neuroblastoma [29], while general cancer risk is increased in DM1 patients [30]. In turn, *SDK1* has been associated with Brugada syndrome marked by an irregular heartbeat [31], with chronic heart disease a DM1 feature [2, 3]. Two notable SVs include an ~6‐kb OGM (PacBio 7,172 bp) *CBFA2T3* deletion (exons 2–12, Figure 2E) and ~6.9‐kb *H3F3A* insertion with PacBio read disruption (Figure 2F) and Illumina short‐read validation (supplementary material, Figure S7C). While the DM1 CTG expansion has been proposed to impact *CBFA2T3* expression and, in turn, dysregulation of downstream target genes, contributing to both neurological and muscular symptoms [32], *H3F3A* encodes a histone protein crucial for forming and maintaining chromatin structure, with mutational dysfunction associated with neurodegenerative disorders, including DM1 [33]. Besides the *CBFA2T3* deletion at VAF 0.25 showing potential for somatic acquisition or clonal haematopoiesis of indeterminate potential (CHIP), a common form of age‐related somatic mosaicism [34], all other VAFs 0.41–0.59 ranged within that expected for inherited heteroplasmy. However, the *CBFA2T3* deletion presented above the inherited threshold (0.3–0.7) in the transmitting father (VAF 0.8), though it showed a normal inheritance fraction (VAF 0.53) in the premutation brother. While no other relevant pathogenic candidates showed this inheritance anomaly, we maintain this SV to be paternally inherited.

Of the seven potentially pathogenic paternally inherited OGM‐derived SVs, besides *H3F3A* unique to proband, both *SPAG16* SVs were shared with DM1 premutation brother and DM1 adult‐onset aunt, *GPHN* and *CBFA2T3* were shared with the brother only, while *CASC15* and *SDK1* were shared by the DM1 and PD phenotypic aunts (Table 2). Paternally inherited gene‐impacting SVs in the brother (supplementary material, Table S2), not shared by the proband yet of potential interest, included a single *VPS13B* ~6.2‐kb insertion shared with both paternal aunts, where deletion events with a second gene hit have been associated with Cohen syndrome marked by numerous developmental and intellectual delays [35]. This is an unlikely contributor in this family. In contrast, three gene‐impacting SVs in the DM1‐aunt sparked further interest (supplementary material, Table S4) and included an ~6‐kb *GMDS* insertion, a gene associated with brain development [36], while the ~10‐kb *GRM8* and ~2‐kb *CSMD1* deletions impact genes associated with an array of neurological disorders [37, 38]. Additionally, the PD‐aunt (Table 3) shared an ~3‐kb *FER1L6* (exon 2) deletion with her siblings (Figure 2G), despite associations of *FER1L6* with distal myopathies that can resemble DM1‐related muscle weakness [39]. She also presented with a private or uniquely inherited ~2.5‐kb exon 19 insertion in *MALRD1* (Figure 2H), reported to be over‐expressed in brain tissue of Alzheimer's disease patients [40], though the functional relevance of this insertion remains uncertain in this context.

### OGM‐derived global copy number variation

While significant global CN variation was not evident in the proband and her direct family, both paternal aunts showed elevated CN loss (supplementary material, Figure S8). For the PD‐aunt, this concurred with recent reports [41]. Most notably, these losses included chr16, chr17, and chr22, which includes 22q11.2, a region which, when deleted, associated with early‐onset PD [42], while complete uniparental loss of chr17 (isodisomy) has been associated with limb‐girdle muscular dystrophy [38].

### PacBio‐derived site‐specific and global methylation probabilities

**Figure 3:**
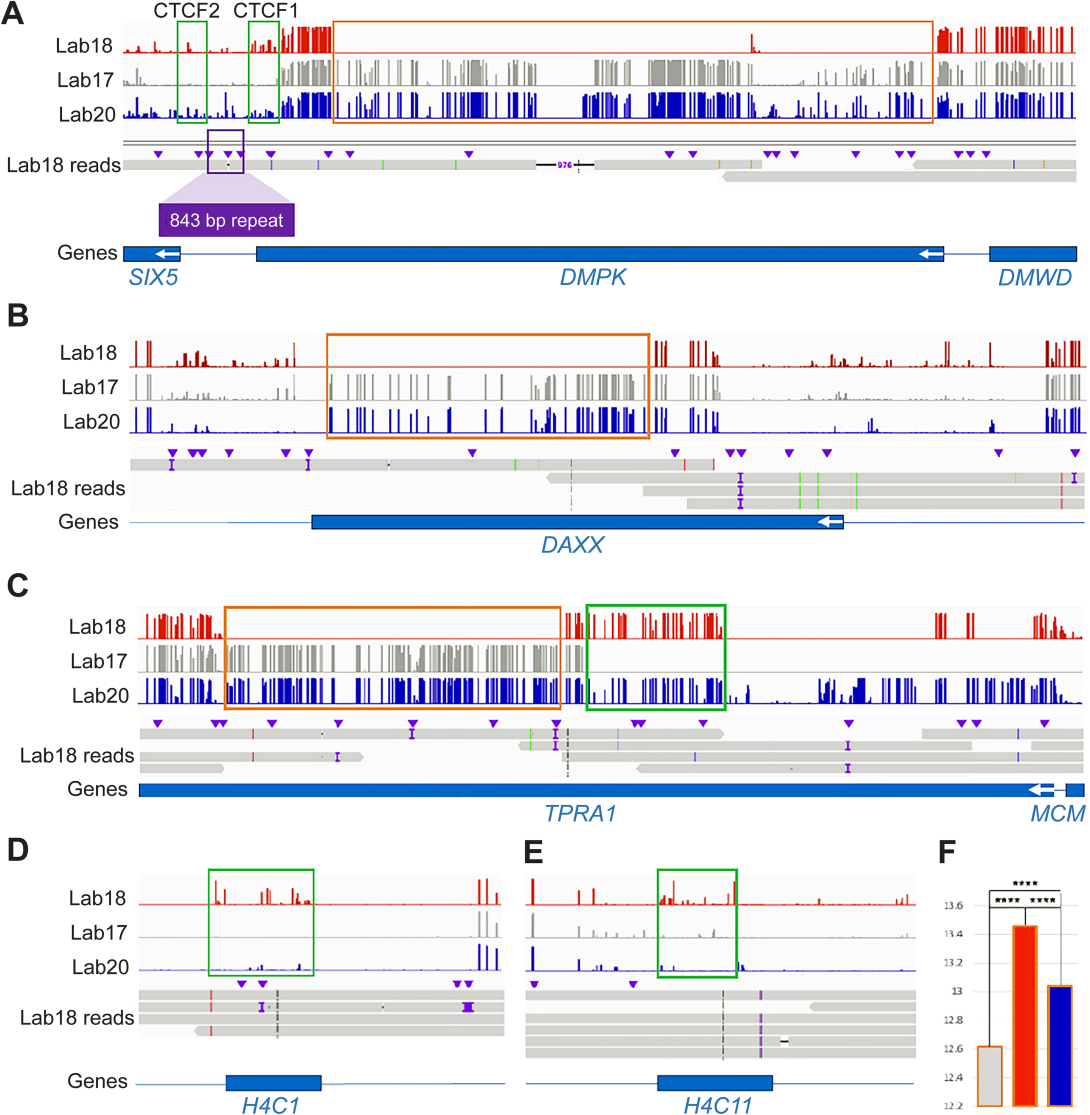
Genome‐wide differential PacBio CpG DNA methylation probabilities from a trio, including the proband with juvenile‐onset DM1 (Lab18 red) and her unaffected mother (Lab17 grey) and transmitting protomutation DM1 father (Lab20 blue). (A) Flanking the DM1 locus, which for Lab18 is defined as an 843‐repeat expansion (purple box), we found the *DMPK* 3’UTR putative CTCF binding sites to be hypermethylated (green boxes) in Lab18 and Lab20 (although CTCF1 is not significant for Lab20), while the coding region of *DMPK* (blue gene band) is notably hypomethylated (orange box) in Lab18 only. Further evidence for proband‐specific gene‐associated hypomethylation included genes involved in histone maintenance, such as (B) *DAXX* and (C) *TRPA1*. The latter gene included a region of hypermethylation shared by the proband and her father, while additional examples of histone maintenance gene‐specific hypermethylation included (D) *H4C1* and (E) *H4C11*. (F) Although in her early 20s, globally Lab18 (red) showed significantly more hypomethylation probability than either parent, triple that of the unaffected mother (Lab 17 grey, early 50s), and double that of her protomutation father (Lab20 blue, mid 50s).

In addition to providing OGM‐derived SV base‐specific validation, the availability of PacBio data allows for further CpG methylation probability assessment. Importantly, DM1 clinical severity, most notably severe congenital presentation, together with longer repeat length and maternal transmission, has been associated with hypermethylation at two CTCF binding sites flanking the repeat region [11, 43]. Intriguingly, the roughly 800‐repeat juvenile‐onset DM1 proband was found to be hypermethylated at both the upstream CTCF1 and downstream CTCF2 binding sites, while her roughly 80‐repeat and largely asymptomatic father showed only CTCF2 hypermethylation probability threshold, and the non‐DM1 mother displayed hypomethylation at both sites (Figure 3A). Additionally, and new to this study, we observed extensive hypomethylation across the entire expanse of the *DMPK* intergenic region in the proband. Again, these observations highlight the atypical presentation in this family, linking *DMPK* hypermethylation to paternal transmission (which is contrary to current assumptions), as well as shorter repeat lengths, including early signs of increased methylation in the protomutation father. While traditionally associated with congenital DM1, it is notable that a study linking methylation status to repeat length suggested a congenital threshold of 653 CTG repeats [11], below that observed for the proband.

As the H3.3 histone plays a critical role in maintaining genomic stability [33], we further examined methylation probability as a consequence of the paternally inherited *H3F3A* insertion. Notable H3.3 interaction players showing proband‐exclusive hypomethylation included an extensive intergenic region impacting the *DAXX* (Figure 3B) and *TPRA1* genes (Figure 3C). Downstream from the H3.3 histone chaperone *MCM2* and immediately upstream from the proband's *TPRA1* hypomethylated block, both proband and father showed hypermethylation. Additional potentially relevant proband hypermethylation was observed spanning the interaction genes *H4C1* (Figure 3D) and *H4C11* (Figure 3E). While genome‐wide CpG sites were overwhelmingly methylated, the proband exhibited ~259,000 and ~94,000 more unmethylated sites compared with the mother (early 50s) and father (mid‐50s) respectively (Figure 3F). Reminiscent of the ageing process [44], the proband (early 20s) showed significant global hypomethylation (all *p* < 0.0001).

## Discussion

It is well established that neuromuscular disorders are highly heterogenous, marked by significant genetic complexity involving often large structural pathogenic variants, including repeat expansions, confounded by epigenetic regulation [45]. While DM1 is a classic repeat expansion neuromuscular disease, PD does not typically fall within the repeat expansion category, although there are notable recent exceptions [46, 47]. However, both are prone to somatic instability, presenting as tissue‐specific acquired variants or age‐related repeat expansion. Although multisystem features are shared, evidence is lacking with regard to shared genetic inheritance. As such, having access to an unusual two‐generation family presenting with varying degrees of DM1 clinical presentation and non‐DM1‐associated early‐onset PD, we sought to examine, using high‐resolution deep‐coverage OGM, shared versus *de novo* global kilo‐to‐megabase‐sized inherited SVs, largely undetectable using traditional methods. The rationale was to reveal a potentially hidden landscape of inherited genomic complexity, both shared and unique within this unusual family with heterogenous DM1 presentation and independent early‐onset PD.

Using more traditional approaches, we estimated DM1 repeat lengths, while confirming two‐generation paternal transmission. Harbouring 13/777 targeted ONT‐predicted or 13/849 PacBio long‐read‐predicted *DMPK* CTG repeat alleles, by the time of diagnosis in her early 20s, the proband presented with a variety of phenotypic characteristics, while her over‐50‐year‐old transmitting protomutation father (12/83 ONT, 12/79 PacBio) remained largely asymptomatic. With significant expansion historically correlated with maternal age and/or adult‐onset symptomatic maternal transmission, attributed to DNA instability during oogenesis [48], we reflected on a study that reported premutation or protomutation males over females were more likely to transmit an expanded repeat [49]. Here, the protomutation paternal transmission in this family unusually resulted in a 9.4‐ (ONT) to 10.8‐fold (PacBio) expansion in the first female offspring (proband) yet an atypical 1.8‐fold ONT‐predicted contraction in the second born premutation brother (100% prevalence). Further reflecting on the high prevalence within this single family (83.3%, 5/6 direct descendants of the transmitting grandfather), it was further noted that the single unaffected aunt (12/16 ONT repeats) presented with early‐onset PD. Using OGM, while confirming *DMPK* repeat pathogenicity in the proband, having set our discovery threshold stringently at >500 bp (as recommended), we were not surprised that no further *DMPK* abnormalities were detected. In contrast to a recent suggestion [18], we were unable to confirm the roughly 534 bp (178 ONT repeats) for the DM1‐aunt, while OGM revealed significant chr19 CN loss. Intriguingly, the non‐DM1 PD‐aunt showed chr19 aneuploidy loss, which requires further consideration with respect to PD presentation.

Globally, while private or *de novo* OGM‐derived SVs were scarce and unlikely to be of clinical relevance, several paternally inherited large‐scale insertions or deletions impacting genes with reported clinical associations were detected in the proband. These included variants corresponding to symptoms either present in the proband, such as tachyarrhythmia associated with *SDK1* mutation/dysfunction [31] and autism‐spectrum disorder associated with *SPAG16* [27] and *GPHN*, given that autism is more prevalent in juvenile and congenital DM1 [50], or DM1‐associated symptoms not presenting in the proband, such as basal cell carcinoma (skin cancer) and other cancers associated with *CASC15* mutation [29]. To the best of our knowledge, no other family members, even those sharing these SVs, have been diagnosed with any or all of these conditions. Notably, *CBFA2T3* encodes for a protein, which when expressed has been shown to be critical for neurogenesis and neuronal differentiation [32]. Though one may speculate that significant deletion of this highly conserved gene may be contributing, at least in part, to phenotypic presentation in the proband and the DM1‐aunt, it is not as evident in the father, and any speculated correlation with repeat length phenotypic predestination requires further investigation. The same is true for the *H3F3A‐*disrupting insertion, shared exclusively between the proband and her transmitting father. This histone encoding gene, directly involved in epigenetic regulation and DNA damage response by facilitating access to the DNA repair machinery, is known to be mutated in neurodegenerative disorders [33].

Overall, and in contrast to maternally inherited or *de novo* (private) large‐scale SVs, while both offspring (proband and brother) were more likely to inherit a paternal over maternal OGM‐derived SV (2.1‐ and 1.44‐fold respectively), paternally inherited SVs were more likely to impact genes associated with neuromuscular conditions (43.8% versus 12.5% and 38.5% versus 0% respectively). Taken together, the third‐generation offspring showed a significant bias towards paternally inherited potentially pathogenic neuromuscular gene‐disrupting SVs (*p* = 0.0158, two‐tailed Fisher's exact test), which increased in significance (*p* = 0.0015) when excluding for the gain rather than loss of function *KANK1* maternally inherited candidate. What remains to be determined is how (and whether) these complex inherited SVs act together, including with *DMPK* repeat length and somatic mosaicism to influence the disparity in clinical manifestations and associated delayed diagnoses within this single family.

Additionally, trio‐based PacBio‐derived methylation probability examination raised further speculation with regard to the contribution of known and unknown global epigenetic modifications. Confirming hypermethylation at both *DMPK* CTCF sites in the symptomatic proband, the non‐carrier mother remained hypomethylated at these sites, and, unique to this study, the asymptomatic protomutation father showed hypermethylation prediction thresholds at CTCF2, with minimal evidence at CTCF1. In contrast to her parents, the proband additionally showed extensive *DMPK* locus hypomethylation. We further report potential repeat expansion‐associated regional methylation differences in known regulators of genomic stability through histone maintenance, including genes such as *DAXX*, *TPRA1*, *H4C1*, and *H4C11*. With the proband in her early 20s, this differential methylation more plausibly reflects secondary consequences of underlying repeat‐associated genomic instability. Limited here to a single trio, we emphasise these are molecular observations rather than definitive quantitative conclusions. A common phenomenon of ageing [44], it is further notable that the young proband showed significant global genome‐wide hypomethylation, double that of her father (mid‐50s) and triple compared to her mother (early 50s). Compounding this observation is the shared *H3F3A‐*disrupting insertion in the proband and father, which calls for further investigation regarding the role of epigenetic regulation in DM1 presentation. Besides the possibility of multiple gene interactions (providing here new gene candidates), together with both local and global epigenetic modification, the potential contribution of chr19‐associated CN loss, as observed for the symptomatic paternally transmitted paternal aunt, has yet to be reported for DM1.

In addition to shared DM1‐phenotypic SVs impacting *SPAG16* and *GPHN*, we provide further speculation with regard to global large‐scale genomic contributions to early‐onset PD. This includes a large deletion of *FERIL6* shown to be associated with distal myopathies [39, 51] and a private insertion impacting *MALRD1*, a gene associated with Alzheimer's disease [40]. With a clear PD rather than DM1 clinical diagnosis and presenting with *DMPK* repeat lengths well within the healthy range (12/16 repeats), OGM profiling suggested chr19 aneuploidy, with notable CN losses across chr16, chr17, and chr22, supporting the growing literature for a role for chromosome‐level inherited abnormalities in PD [41, 42]. Although sharing numerous symptoms and associated disease course, DM1 and PD have yet to be linked genetically. Here in a single family with independent DM1 and PD presentation, we provide an as yet unseen global view of inherited over acquired, shared, and DM1/PD‐specific genomic complexity, including a non‐random skewing towards potential pathogenic relevance.

The limitations of our study include our inability to directly link potential pathogenic large‐scale SVs with clinical presentation, including associated functionality thorough model systems. While OGM is not a sequencing but rather a spatial technology, pathogenic candidates lack base‐specific resolution. As such, we used low‐coverage long‐read (PacBio) and deep‐coverage short‐read (Illumina) inspections to verify 100% and 57% proband potentially pathogenic SVs, respectively. While this study was not aimed at providing technology comparisons, each with their specific advantages and limitations, when matched for cost, OGM generates the deepest coverage (60×), with SV confidence requiring in this study a minimum of five DNA molecules, followed by Illumina (30×) and lowest for PacBio (10×). However, it is well established that low‐coverage PacBio data have higher call accuracy for pathogenic SVs than cost‐matched short‐read Illumina sequencing [52]. However, low‐coverage proband‐parental trio‐derived PacBio data proved sufficient to achieve SV validations. Besides a lack of functional validation for potential pathogenic SVs, due to the more recent adoption of OGM, the limitation of the OGM‐derived SV database to 270 largely European ancestral healthy controls raises additional restrictions. This is, however, diminished as the family in question is of European ancestry. In turn, the lack of reported DM1 and PD presentation within a single family limits opportunities for between‐family validations. Having access to OGM‐derived data generated for multi‐generational DM1 and PD families will provide an opportunity for clinically relevant validations.

## Conclusions

In this study, we applied high‐resolution whole‐genome imaging to characterise the complete landscape of inherited and *de novo* large‐scale genomic variance in a single family with two generational DM1 presentation, including atypical intergenerational repeat dynamics and a single case of non‐DM1‐associted PD. In contrast to the asymptomatic permutational brother, the classical to severe DM1 proband inherited a 1.5‐fold greater overall fraction of paternal over maternal large (> 1,000 bp) genome‐wide SVs. Notably, for the paternally inherited gene‐impacting SVs, the proband showed a 1.3‐fold increased fraction for genes associated with neuromuscular function. Sharing different combinations of notable gene‐impacted candidates across the range of DM1‐associated clinical presentations within the family, while we were unable to make any direct clinical correlations, it remains to be elucidated whether these complex inherited genomic abnormalities act synergistically, contributing (at least partially) to variability in genetic anticipation. Overall, having uncovered a higher level of inherited structural and epigenetic complexity beyond the primary repeat locus in this unusual DM1 family, we highlighted opportunities for further investigations into the clinical significance of large‐scale global disruptions across the broad spectrum of neuromuscular disorders.

## Author contributions statement

VMH designed the study. MMH and VMH acquired the funding. MMH and RJL performed the formal OGM analysis, JC the differential methylation analysis, TG and MMH the short‐read sequencing validation analysis, IWD, IS and SRC the ONT‐targeted long‐read analysis, and MMH, WJ and VMH the PacBio long‐read analyses. MMH, JC, KRK and VMH led the data interpretation. KRK provided clinical review, ethical consent, recruitment and clinical interpretation. VMH supervised the study. MMH and VMH wrote the manuscript and generated the figures. All authors read and approved the final manuscript.

## Supporting information


**Figure S1.** Diagnostic ONT *DMPK*‐targeted screening in proband and extended family members
**Figure S2**. OGM‐derived all large variant type circos plots with a focus on chr19 in proband and extended family members
**Figure S3**. IGV‐guided short‐read *DMPK* locus repeat‐associated breakend validation in proband
**Figure S4**. OGM‐derived whole‐genome and all large variant type circos plots in proband and extended family members
**Figure S5**. Total counts of OGM‐derived whole‐genome structural variant types in proband and extended family members
**Figure S6**. OGM‐revealed and long‐and‐short‐read validated inherited and acquired chr9p gains and losses impacting the proband
**Figure S7**. IGV‐guided short‐read validation of OGM‐derived structural variants
**Figure S8**. OGM‐derived genome‐wide copy number gains and losses for the DM1 protomutation father and his early‐onset PD and adult‐onset classical DM1 siblings
**Table S1**. Optical genome mapping (OGM) summary statistics and structural variant (SV) calls
**Table S2**. Unique optical genome mapping (OGM)‐derived structural variants (SVs, *n* = 23) identified in younger premutation brother (Lab19)
**Table S3**. Unique optical genome mapping (OGM)‐derived structural variants (SVs, *n* = 30) identified in father (Lab20)
**Table S4**. Unique optical genome mapping (OGM)‐derived structural variants (SVs, *n* = 33) identified in DM1‐presenting paternal aunt (Lab22)

## Data Availability

The datasets used and/or analysed during this study are available from the corresponding author on reasonable request.
