# Supplementary material for: Optical mapping reveals a higher level of large‐scale structural variants in a family with paternally transmitted myotonic dystrophy and independent Parkinson's disease
Source: J Pathol. 2026 Jun 9;270(1):83–97. doi: 10.1002/path.70084 (PMC13431841; doi:10.1002/path.70084)
Supplement: Supplementary file 1 — Figure S1. Diagnostic ONT DMPK‐targeted screening in proband and extended family members Figure S2. OGM‐derived all large variant type circos plots with a focus on chr19 in proband and extended family members Figure S3. IGV‐guided short‐read DMPK locus repeat‐associated breakend validation in proband Figure S4. OGM‐derived whole‐genome and all large variant type circos plots in proband and extended family members Figure S5. Total counts of OGM‐derived whole‐genome structural variant types in proband and extended family members Figure S6. OGM‐revealed and long‐and‐short‐read validated inherited and acquired chr9p gains and losses impacting the proband Figure S7. IGV‐guided short‐read validation of OGM‐derived structural variants Figure S8. OGM‐derived genome‐wide copy number gains and losses for the DM1 protomutation father and his early‐onset PD and adult‐onset classical DM1 siblings Table S1. Optical genome mapping (OGM) summary statistics and structural variant (SV) calls Table S2. Unique optical genome mapping (OGM)‐derived structural variants (SVs, n = 23) identified in younger premutation brother (Lab19) Table S3. Unique optical genome mapping (OGM)‐derived structural variants (SVs, n = 30) identified in father (Lab20) Table S4. Unique optical genome mapping (OGM)‐derived structural variants (SVs, n = 33) identified in DM1‐presenting paternal aunt (Lab22) [file PATH-270-83-s001.docx]

**Optical mapping reveals a higher level of large-scale structural variants in a family with paternally transmitted myotonic dystrophy and independent Parkinson’s disease**

Md. Mehedi Hasan *et al. J Pathol* <https://doi.org/10.1002/path.70084>

Supplementary Figures S1–S8

Supplementary Tables S1–S4

**
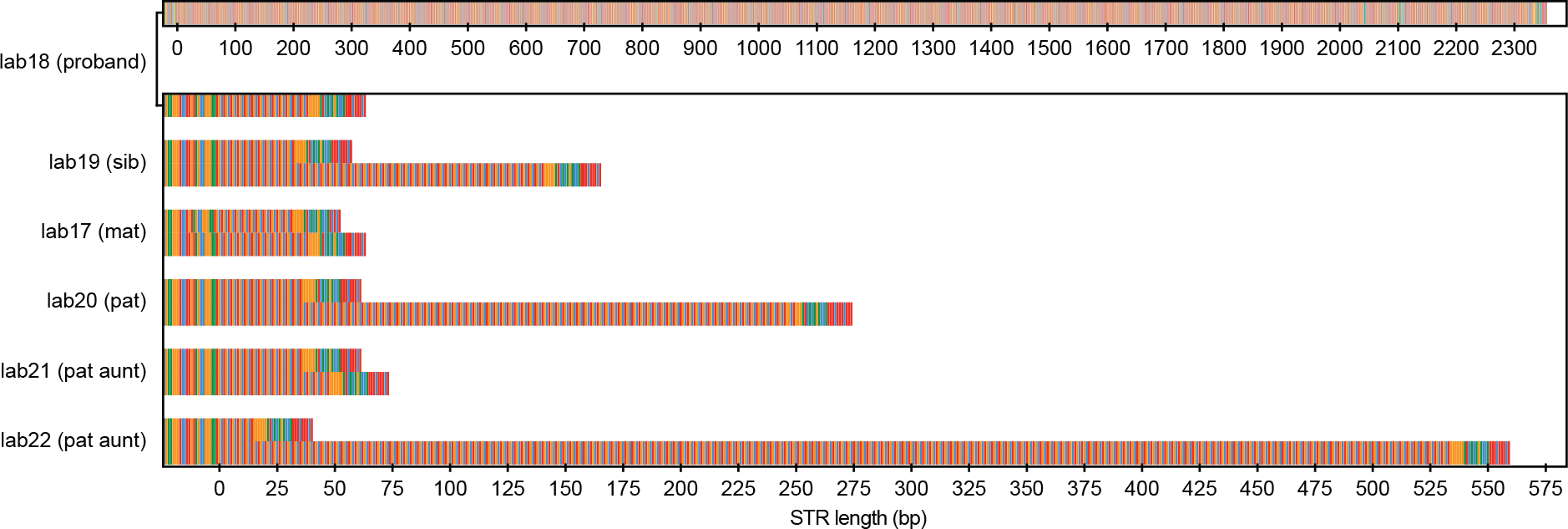
**

**Figure S1**. **Diagnostic ONT *DMPK*-targeted screening in proband and extended family members.** Diagnostic ONT-targeted screening representing uninterrupted *DMPK* CTG repeat lengths for the proband (Lab18, 13/777 repeats) and family members including the brother (Lab19, 11/47), mother (Lab17, 11/13), father (Lab20, 12/83), and paternal aunts with PD (Lab21, 12/16) or clinically confirmed DM1 (Lab22, 5/178). The nucleotide bases are coloured as follows: red = T, blue = C, green = A, orange = G.


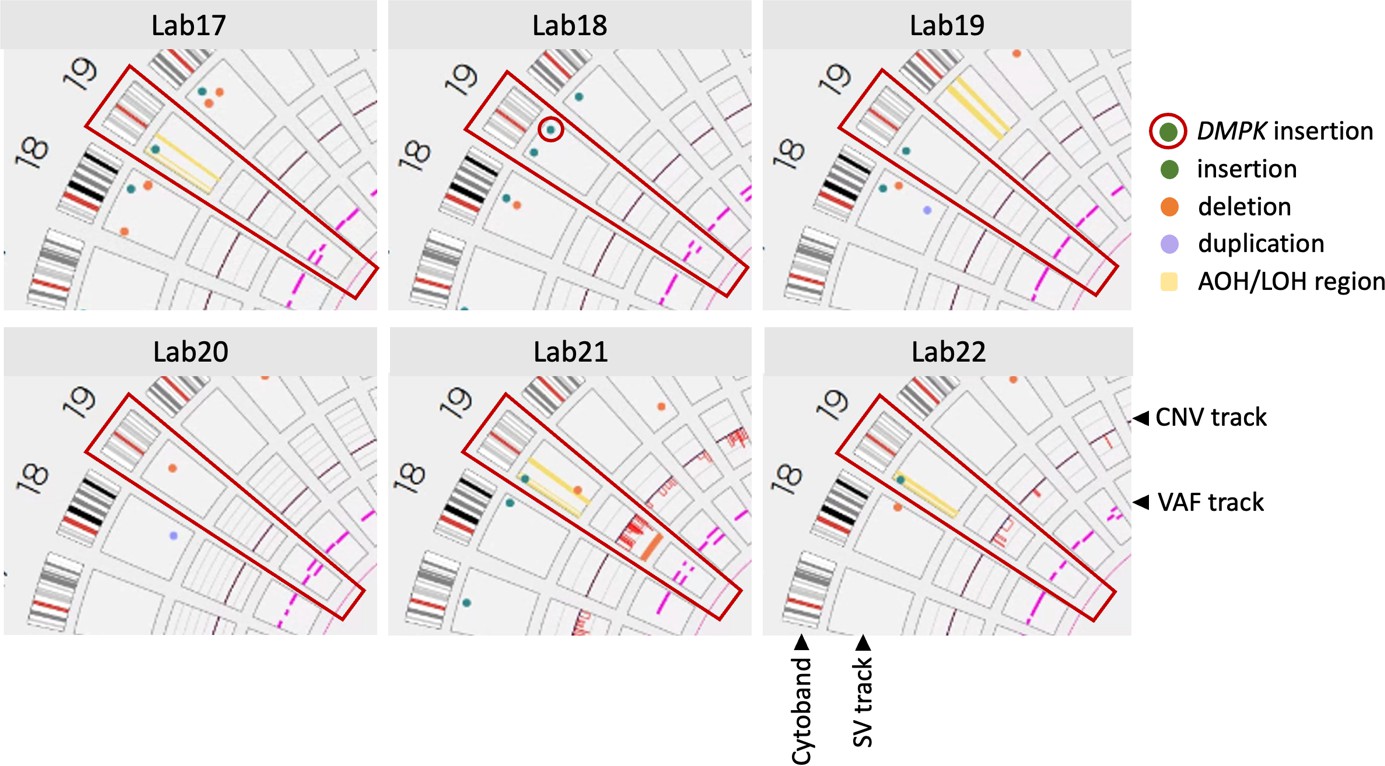


**Figure S2**. **OGM-derived all large variant type circos plots with a focus on chr19 in proband and extended family members**. OGM circos plots highlighting SV by type and CNV with a focus on chr19 germline DNA from study proband (Lab18), including OGM-inferred *DMPK* expansion and proband’s mother (Lab17), DM1 premutation brother (Lab19), DM1 protomutation father (Lab20), and paternal aunts with PD (Lab21) and DM1 (Lab22) respectively.


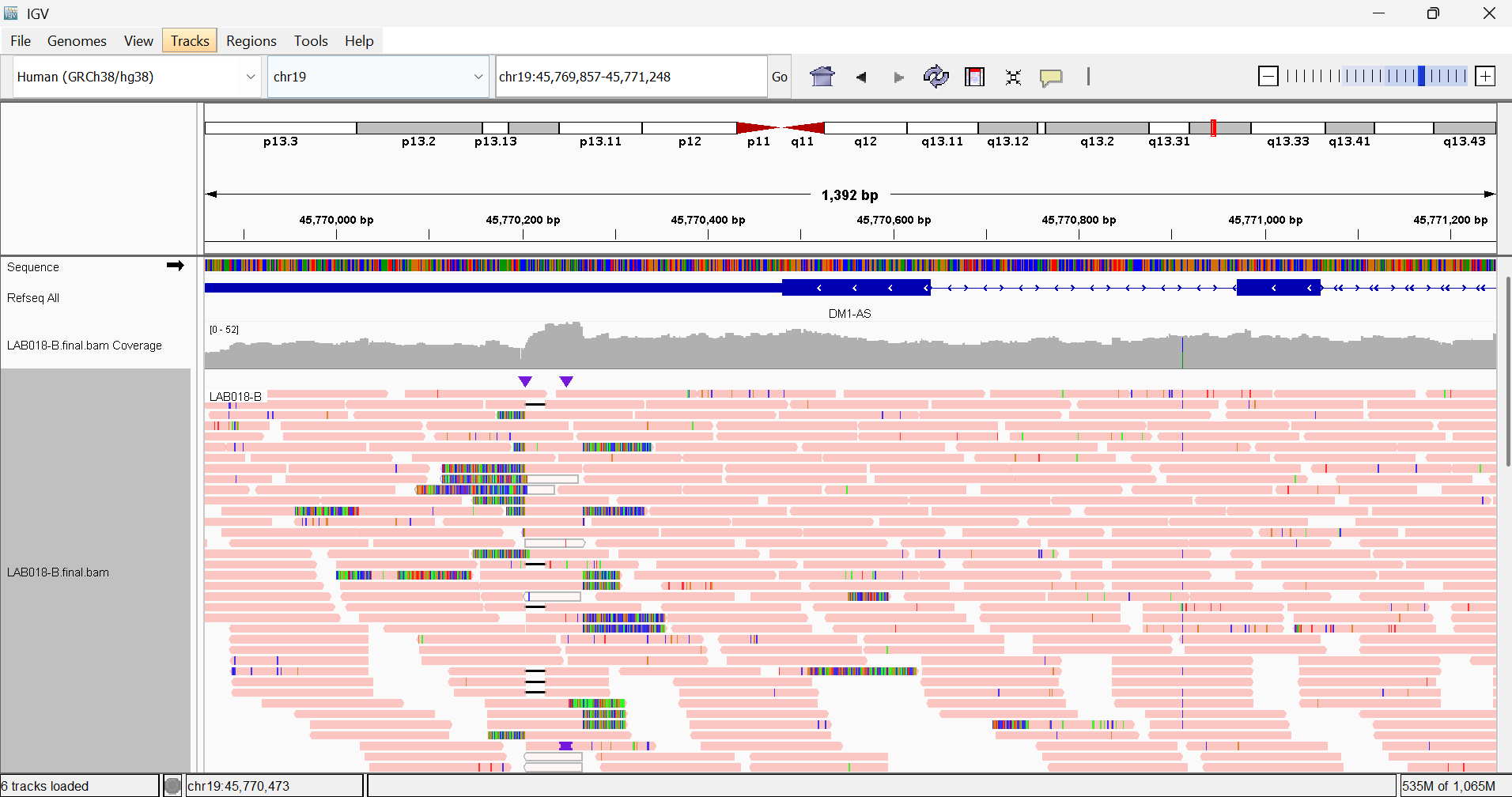


**Figure S3**. **IGV-guided short-read *DMPK* locus repeat-associated breakend validation in proband.** IGV-guided manual inspection for Lab18 Illumina short reads upstream of *DMPK* locus on chr19 (45,769,857–45,771,248; GRCh38 reference) confirming repeat-associated breakends (purple triangles).


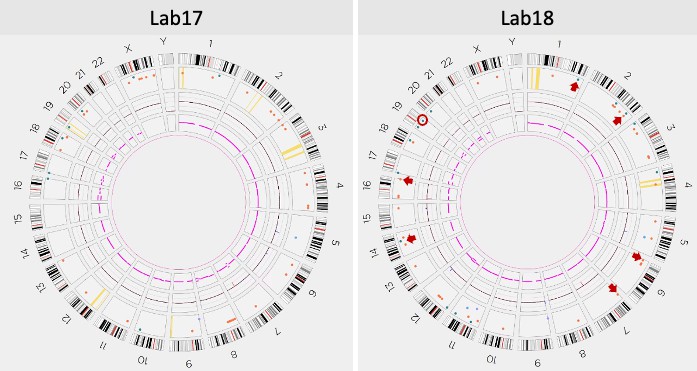


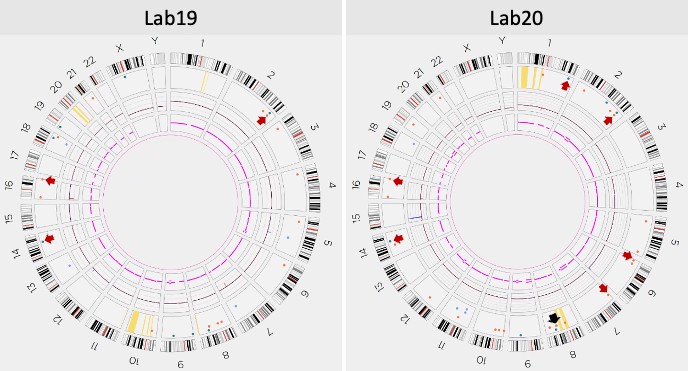

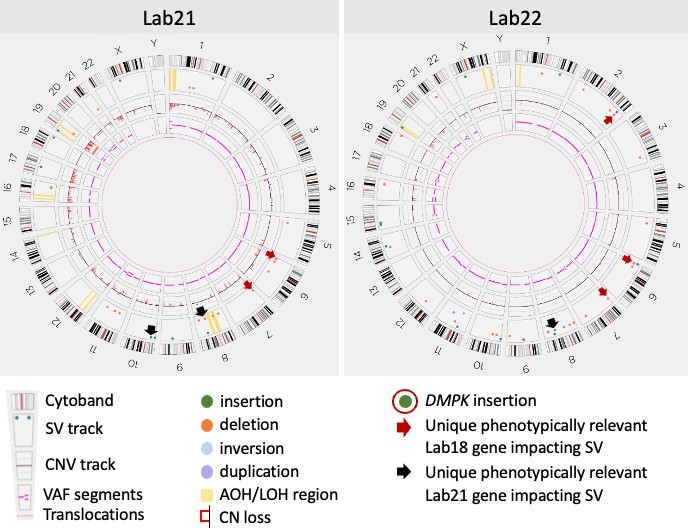


**Figure S4**. **OGM-derived whole-genome and all large variant type circos plots in proband and extended family members**. OGM circos plots depicting unique SVs by type and location, CN losses (red) and gains (blue), and VAF for juvenile-onset DM1 proband (Lab18) and respective family members including her unaffected mother (Lab17), premutation brother (Lab19), protomutation father (Lab20), paternal aunt with early-onset PD (Lab21), and adult-onset DM1 paternal aunt (Lab22). Potential clinically relevant SVs (green insertions, orange deletions) impacting notable candidate genes in Lab18 (red arrows) are all paternally inherited, while two additional candidate SVs were observed in Lab21 (black arrows), with co-inheritance between family members further depicted.


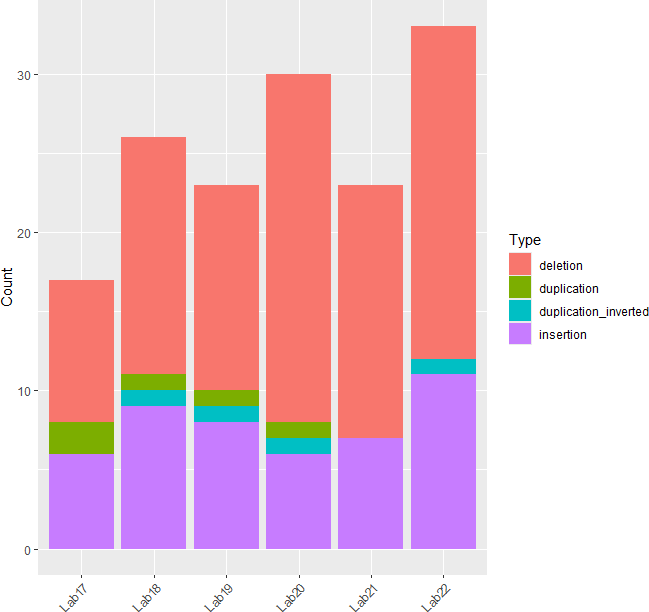


**Figure S5**. **Total counts of OGM-derived whole-genome structural variant types in proband and extended family members.** Distribution (total count) of genome-wide OGM-derived SVs by type in proband (LAB18) and extended family members including mother (Lab17), premutation sibling (Lab19), protomutation father (Lab20), PD-aunt (Lab21), and DM1-aunt (Lab22).


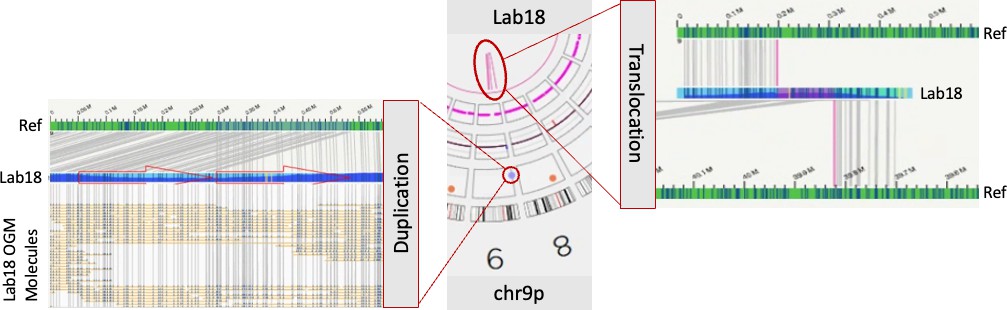


**A**

#
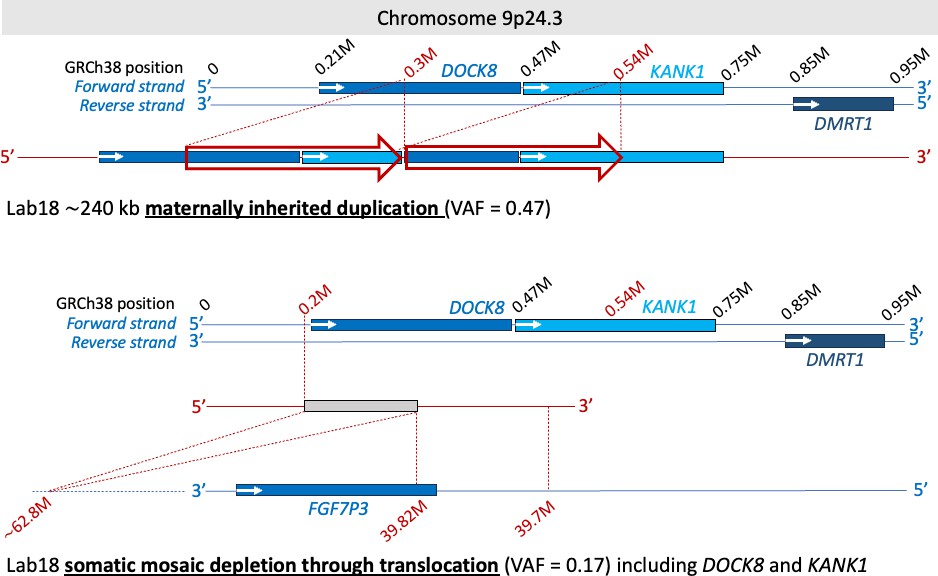
B

**C**


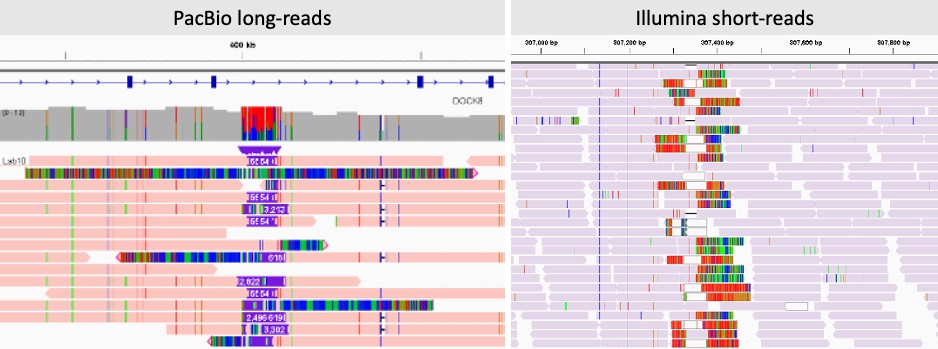


**Figure S6**. **OGM-revealed and long- and short-read validated inherited and acquired chr9p gains and losses impacting proband.** (A) Detected using OGM a maternally inherited duplication (left inset) and somatically acquired translocation (right inset, under less stringent conditions) impacting *DOCK8* and *KANK1* genes were identified in proband (Lab18 circos plot snapshot) and in chr 9p24.3 region of known relevance to neuromuscular disorders. (B) Schematic representation, including gene positions and orientation, for maternally inherited duplication (top) and somatic translocation (bottom) identified in proband. (C) IGV manual inspection for PacBio long-read (left) and Illumina short-read (right) breakpoint validations for maternally inherited duplication within *DOCK8*.


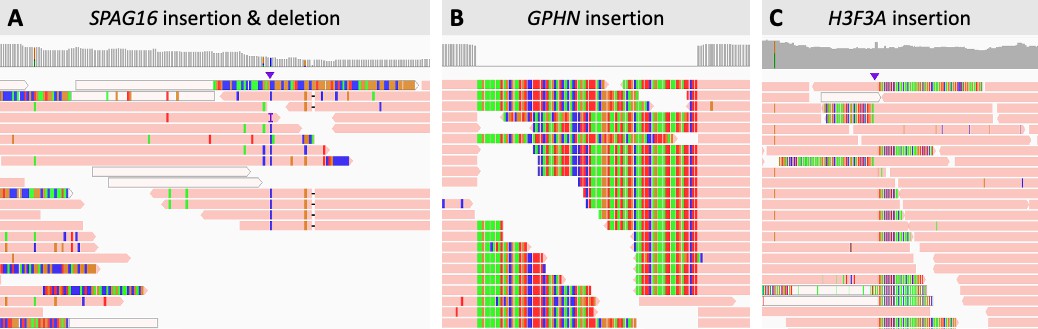


**Figure S7**. **IGV-guided short-read validation of OGM-derived structural variants**. IGV validation of OGM-derived structural variation breakpoints using Illumina short-read manual inspection for paternally inherited large SVs impacting potential pathogenic candidate genes including (A) *SPAG16* (two SVs) and (B) *GPHN* and *H3F3A*.


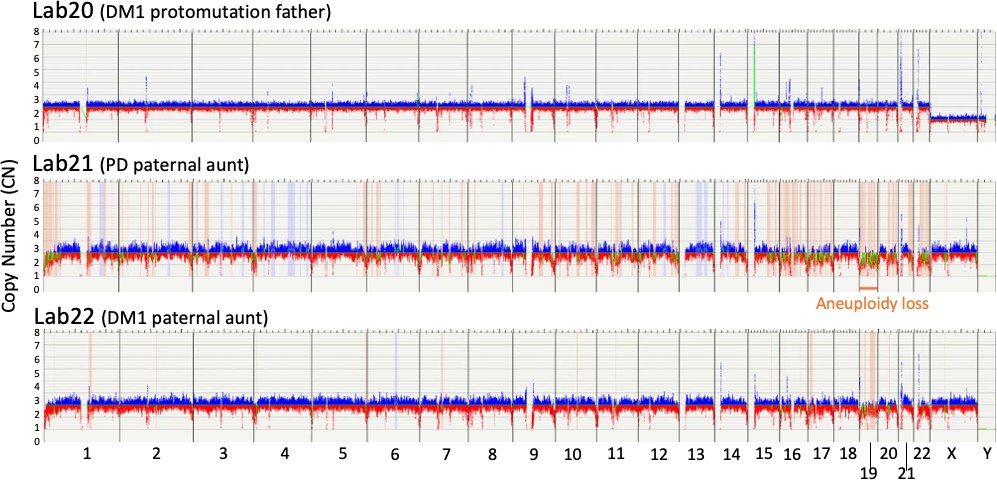


**Figure S8**. **OGM-derived genome-wide CN gains (blue) and losses (red) for early-onset PD paternal aunt (Lab21) and DM1 protomutation (Lab20) and adult-onset classical DM1 (Lab22) siblings.**

**Table S1.** Optical genome mapping (OGM) summary statistics and structural variant (SV) calls.

|  | **Lab17** | **Lab18** | **Lab19** | **Lab20** | **Lab21** | **Lab22** |
| --- | --- | --- | --- | --- | --- | --- |
| DM1 family position | Mother | Proband | Brother | Father | Pat Aunt | Pat Aunt |
| **OGM molecule summary statistics** | | | | |  |  |
| Total number of molecules | 1,799,051 | 1,548,950 | 2,194,272 | 3,074,260 | 2,324,503 | 1,376,364 |
| Total length (Mbp) | 421,799.99 | 382,518.86 | 626,865.95 | 754,028.26 | 666,284.88 | 378,489.48 |
| Average length (kbp) | 234.46 | 246.95 | 285.68 | 245.27 | 286.64 | 274.99 |
| Molecule N50 (kbp) | 227.17 | 247.41 | 295.33 | 242.82 | 300.04 | 285.81 |
| Label density per 100 kb | 15.72 | 15.71 | 14.87 | 15.71 | 9.96 | 13.79 |
| Coverage of reference | 136.58 × | 123.86 × | 202.98 × | 244.16 × | 215.75 × | 122.56 × |
| Total DNA ≥ 150 kbp, ≥ 9 DLE sites per Gbp | 425.05 | 392.16 | 764.41 | 797.9 | 1,732.94 | 1,711.01 |
| Total number of molecules aligned | 1,365,370 | 1,155,089 | 1,708,745 | 2,885,171 | 931,772 | 959,147 |
| Effective coverage | 83.54 × | 83.68 × | 170.29 × | 224.26 × | 76.13 × | 77.11 × |
| **OGM *de novo* assembly statistics** | | | | |  |  |
| Total reference length (Mbp) | 3,088.27 | 3,088.27 | 3,088.27 | 3,088.27 | 3,088.27 | 3,088.27 |
| Total number of genome maps aligned | 658 | 567 | 557 | 579 | 460 | 536 |
| Total unique aligned length (Mbp) | 2,838.32 | 2,843.49 | 2,860.56 | 2,865.79 | 2,836.76 | 2,835.64 |
| Total unique aligned length/reference length | 0.92% | 0.92% | 0.93% | 0.93% | 0.92% | 0.92% |
| Total number of molecules aligned | 1,345,002 | 1,146,038 | 1,698,457 | 2,885,171 | 975,772 | 976,257 |
| **OGM-called SV summary and AOH/LOH (pre-filtering)** | | | | |  |  |
| Deletions | 1,329 | 1,365 | 1,268 | 1,238 | 1,157 | 1,189 |
| Insertions | 2,664 | 2,725 | 2,831 | 2,765 | 2,692 | 2,730 |
| Duplications | 52 | 61 | 77 | 82 | 56 | 48 |
| Inversion breakpoints | 58 | 59 | 65 | 61 | 63 | 45 |
| Interchr: translocation breakpoints | 0 | 0 | 0 | 0 | 0 | 0 |
| Interchr: fusion breakpoints | 0 | 0 | 3 | 2 | 1 | 3 |
| Total SVs | 5,103 | 5,210 | 5,244 | 5,148 | 3,969 | 4,015 |
| Fraction of autosomal AOH/LOH | 0.023 | 0.014 | 0.024 | 0.026 | 0.033 | 0.006 |

AOH, absence of heterozygosity; DM1, myotonic dystrophy type 1; Gbp, gigabase pairs; LOH, loss of heterozygosity; Mbp, megabase pairs; OGM, optical genome mapping; SV, structural variants.

**Table S2.** Unique optical genome mapping (OGM)-derived structural variants (SVs, *n* = 23) identified in the younger premutation brother (Lab19).

| **Chromosome: position** | **SV type** | **Size (bp)** | **VAF** | **Overlapping genes**1 | **Nearest non-overlapping locus**2 | **Co-inherited** |
| --- | --- | --- | --- | --- | --- | --- |
| **Private SVs (*n*= 1)** | | | | | | |
| chr13:52505311–52712693 | DUP | 207,383 | 0.41 | *AL137058.1, MRPS31P4, AL137058.2, HNRNPA1L2, AL139089.1, SUGT1, CNMD* | *AL137058.3* | NA |
| **Paternally inherited SVs (*n*= 13)** | | | | | | |
| chr2:187671166–187685624 | DEL | 12,819 | 0.4 | - | *LINC01090* | proband, DM1-aunt |
| chr2:213559670–213572659 | DEL | 6,050 | 0.42 | ***SPAG16*** | *MIR4438* | proband, DM1-aunt |
| chr2:213504200–213513768 | INS | 3,444 | 0.4 | ***SPAG16*** | *SPAG16-DT* | proband, DM1-aunt |
| chr8:93158852–93166540 | DEL | 4,545 | 0.61 | *C8orf87* | *LINC00535* | PD-aunt, DM1-aunt |
| chr8:93961953–93965700 | DEL | 1,119 | 0.49 | *-* | *RPL34P18* | PD-aunt, DM1-aunt |
| chr8:99845541–99859871 | INS | 6,203 | 0.48 | ***VPS13B*** | *COX6C* | PD-aunt, DM1-aunt |
| chr9:86985023–86995208 | INS | 6,000 | 0.4 | *GAS1RR* | *AL513318.2* | PD-aunt |
| chr10:22008378–22014142 | DEL | 4,346 | 0.59 | - | *DNAJC1* | DM1-aunt |
| chr14:66947112–66959944 | INS | 2,056 | 0.41 | ***GPHN*** | *AL049835.1* | Proband |
| chr16:8092039–8340701 | DEL | 211,822 | 0.52 | *AC093515.1, AC018767.2, LINC02152, AC018767.3* | *AC018767.1* | Proband |
| chr16:88868384–88914625 | DEL | 6,264 | 0.53 | ***CBFA2T3,*** *AC092384.2* | *PABPN1L* | Proband |
| chr18:61006158–61020756 | DUP | 14,599 | 0.54 | - | *AC113137.1* | - |
| chr21:32251481–32266664 | DEL | 5534 | 0.58 | *AP000265.1* | *MIS18A* | PD-aunt, DM1-aunt |
| **Maternally inherited SVs (*n*= 9)** | | | | | | |
| chr3:24848468–24867812 | DEL | 16,647 | 0.5 | *AC092422.1* | *RN7SL216P* | proband |
| chr3:24848468–24867812 | DEL | 16,647 | 0.5 | *AC092422.1* | *RN7SL216P* | proband |
| chr6:26727983–26750048 | DEL | 8,011 | 0.49 | - | *AL513548.1* | proband |
| chr8:34483569–34489210 | DEL | 1,438 | 0.61 | - | *AC090993.1* | proband |
| chr18:57566156–57574292 | INS | 4,025 | 0.57 | *FECH* | *AC100847.1* | - |
| chr18:70883312–70900658 | DEL | 2,078 | 0.59 | *-* | *AC090415.2* | - |
| chr19:10341841–10346616 | INS | 2,076 | 0.46 | - | *ICAM3* | proband |
| chrX:57999084–58012717 | INS | 11,147 | 1 | - | *KRT8P17* | - |

1Genes in bold indicate neuromuscular-related gene candidates. 2Not all entries represent official HUGO gene symbols. Some loci correspond to ENSEMBL-predicted transcripts or gene models that require experimental confirmation.

DEL, deletion; DM1, myotonic dystrophy type 1; INS, insertion; OGM, optical genome mapping; PD, Parkinson’s disease; SV, structural variant; VAF, variant allele frequency.

**Table S3.** Unique optical genome mapping (OGM) derived structural variants (SVs, *n* = 30) identified in the father (Lab20).

| **Chromosome: position** | **SV type** | **Size (bp)** | **VAF** | **Overlapping genes**1 | **Nearest non-overlapping locus**2 | **Co-inherited** |
| --- | --- | --- | --- | --- | --- | --- |
| **Non-sibling shared SVs (*n* = 9)** | | | | | | |
| chr1:226071312–226074381 | INS | 6,794 | 0.57 | ***H3F3A*** | *LINC01703* | Proband |
| chr2:151197448–151212963 | INS | 6,155 | 0.56 | - | *FABP5P10* | Proband |
| chr5:167222912–167245218 | DEL | 4,969 | 0.4 | - | *TENM2* | - |
| chr10:123041–131734 | DEL | 2,732 | 0.29 | - | *ZMYND11* | - |
| chr1:113243421–113260985 | DEL | 3,801 | 0.49 | - | *AL357055.2* | Proband |
| chr14:66944487–66959894 | INS | 1,975 | 0.47 | ***GPHN*** | *AL049835.1* | Proband, brother |
| chr16:8092039–8340701 | DEL | 211,741 | 0.51 | *AC093515.1, AC018767.2; LINC02152, AC018767.3* | *AC018767.1* | Proband |
| chr16:88868384–88914625 | DEL | 6,217 | 0.8 | ***CBFA2T3,*** *AC092384.2* | *PABPN1L* | Proband |
| chr18:61006158–61020756 | DUP | 14,599 | 0.5 | - | *AC113137.1* | Brother |
| **Sibling shared SVs (*n* = 21)** | | | | | | |
| chr2:187671166–187685624 | DEL | 12,769 | 0.56 | - | *LINC01090* | Proband, brother, DM1-aunt |
| chr2:213559670–213572659 | DEL | 6,082 | 0.67 | ***SPAG16*** | *MIR4438* | Proband, brother, DM1-aunt |
| chr2:213504200–213513768 | INS | 3,511 | 0.57 | ***SPAG16*** | *SPAG16-DT* | Proband, brother, DM1-aunt |
| chr2:39629742–39641022 | DEL | 3,255 | 0.39 | NA | *TMEM178A* | DM1-aunt |
| chr6:21888943–21900011 | DEL | 4,888 | 0.45 | *CASC15* | *AL136313.1* | Proband, PD-aunt, DM1-aunt |
| chr6:5963134–5972148 | DEL | 1,092 | 0.64 | - | *PKMP5* | PD-aunt |
| chr7:3567199–3605371 | DEL | 8,562 | 0.41 | ***SDK1*** | *AC011284.1* | Proband, PD-aunt, DM1-aunt |
| chr8:99845541–99859871 | INS | 6,211 | 0.42 | ***VPS13B*** | *COX6C* | Brother, PD-aunt, DM1-aunt |
| chr8:123946595–123956695 | DEL | 3,078 | 0.4 | ***FER1L6*** | *AC090753.1* | PD-aunt, DM1-aunt |
| chr8:124724677–124749262 | DEL | 1,416 | 0.49 | *MTSS1* | *AC100858.3* | PD-aunt, DM1-aunt |
| chr8:93158852–93166540 | DEL | 4,582 | 0.49 | *C8orf87* | *LINC00535* | Brother, PD-aunt, DM1-aunt |
| chr8:93961953–93965700 | DEL | 1,139 | 0.4 | - | *RPL34P18* | Brother, PD-aunt, DM1-aunt |
| chr9:86985010–86995221 | INS | 5,982 | 0.73 | *GAS1RR* | *AL513318.2* | Brother, PD-aunt |
| chr10:22008378–22014142 | DEL | 4,326 | 0.48 | - | *DNAJC1* | Brother, DM-aunt |
| chr10:38609506–38620296 | DEL | 2,033 | 0.53 | *AL133173.2* | *ABCD1P2* | DM1-aunt |
| chr10:38609506–38620296 | DEL | 2,071 | 0.53 | *AL133173.2* | *ABCD1P2* | DM1-aunt |
| chr11:37762260–37818524 | DUP | 56,265 | 0.52 | - | *RPL7AP56* | Proband, DM1-aunt |
| chr12:54304003–54321540 | DEL | 10,453 | 0.62 | *AC078778.1, RNU6-950P* | *NFE2* | Proband, DM1-aunt |
| chr14:46540452–46556096 | DEL | 4,344 | 0.36 | - | *LINC00871* | Proband, DM1-aunt |
| chr19:32733748–32743329 | DEL | 5,275 | 0.36 | *TDRD12* | *AC008736.2* | DM1-aunt |
| chr21:32251481–32266664 | DEL | 5,546 | 0.64 | *AP000265.1* | *MIS18A* | Brother, PD-aunt, DM1-aunt |

1Genes in bold indicate neuromuscular-related gene candidates. 2Not all entries represent official HUGO gene symbols. Some loci correspond to ENSEMBL-predicted transcripts or gene models that require experimental confirmation. DEL, deletion; DM1, myotonic dystrophy type 1; INS, insertion; OGM, optical genome mapping; PD, Parkinson’s disease; SV, structural variant; VAF, variant allele frequency.

**Table S4**. Unique optical genome mapping (OGM)-derived structural variants (SVs, *n*= 33) identified in the DM1-presenting paternal aunt (Lab22).

| **Chromosome: position** | **SV type** | **Size (bp)** | **VAF** | **Overlapping genes**1 | **Nearest non-overlapping locus**2 | **Co-inherited** |
| --- | --- | --- | --- | --- | --- | --- |
| **Non-sibling shared SVs (*n* = 8)** | | | | | | |
| chr6:2032400–2049585 | INS | 5,921 | 0.43 | ***GMDS*** | *AL035693.1* | NA |
| chr7:127159287–127171346 | DEL | 9,981 | 0.42 | ***GRM8*** | *AC000099.1* | NA |
| chr8:4863380–4874736 | DEL | 1,715 | 0.44 | ***CSMD1*** | *PAICSP4* | NA |
| chr9:125791574–125818285 | DEL | 8,001 | 0.49 | *PBX3* | *AL358074.1* | NA |
| chr9:66296472–66313745 | INS | 2,030 | 0.04 | - | *AL591479.1* | NA |
| chr15:20628640–20689014 | INS | 1,802,601 | 0.93 | *IGHV1OR15-6, AC023310.4, NBEAP1, AC131280.1* | *AC023310.2* | NA |
| chr15:20628640–20689014 | INS | 1,805,631 | 0.93 | *IGHV1OR15,6, AC023310.4, NBEAP1, AC131280.1* | *AC023310.2* | NA |
| chr15:28372602–28377096 | INS | 14,917 | 0.65 | - | *GOLGA8F* | NA |
| **Sibling shared SVs (*n*= 25)** | | | | | | |
| chr2:187671166–187685624 | DEL | 12,842 | 0.51 | - | *LINC01090* | Proband, brother, father-proband |
| chr2:213566756–213578188 | DEL | 6,054 | 0.52 | ***SPAG16*** | *MIR4438* | Proband, brother, father-proband |
| chr2:39629742–39641022 | DEL | 3,295 | 0.46 | - | *TMEM178A* | Father-proband |
| chr2:213504200–213513768 | INS | 3,474 | 0.56 | ***SPAG16*** | *SPAG16-DT* | Proband, brother, father-proband |
| chr6:21889122–21901847 | DEL | 4,309 | 0.64 | ***CASC15*** | *AL136313.1* | Proband, father-proband, PD-aunt |
| chr7:3558438–3576177 | DEL | 9,048 | 0.41 | ***SDK1*** | *AC011284.1* | Proband, father-proband, PD-aunt |
| chr8:123946595–123956695 | DEL | 3,038 | 0.45 | ***FER1L6*** | *AC090753.1* | Father-proband, PD-aunt |
| chr8:124724677–124749262 | DEL | 1,489 | 0.57 | *MTSS1* | *AC100858.3* | Father-proband, PD-aunt |
| chr8:24843835–24856561 | DEL | 3,052 | 0.48 | - | *AF106564.1* | PD-aunt |
| chr8:93158852–93166540 | DEL | 4,577 | 0.5 | *C8orf87* | *LINC00535* | Brother, father-proband, PD-aunt |
| chr8:93960548–93965700 | DEL | 1,008 | 0.47 | - | *RPL34P18* | Brother, father-proband, PD-aunt |
| chr8:99845541–99859871 | INS | 6,121 | 0.55 | ***VPS13B*** | *COX6C* | Brother, father-proband, PD-aunt |
| chr10:22010172–22035051 | DEL | 4,271 | 0.36 | - | *DNAJC1* | Brother, father-proband |
| chr10:38609506–38620296 | DEL | 2,097 | 0.44 | *AL133173.2* | *ABCD1P2* | Father-proband |
| chr10:2271509–2279763 | INS | 6,199 | 0.46 | - | *LINC00701* | PD-aunt |
| chr11:37738303–37762260 | INS | 77,234 | 0.48 | - | *RPL7AP56* | Proband |
| chr11:57957418–57967013 | DEL | 1,171 | 0.6 | - | *OR5BD1P* | Proband |
| chr11:37762260–37818524 | DUP | 56,265 | 0.49 | - | *RPL7AP56* | Proband, brother, father-proband |
| chr12:54304003–54321540 | DEL | 10,485 | 0.49 | *AC078778.1, RNU6-950P* | *NFE2* | Proband, father-proband |
| chr14:46540452–46556096 | DEL | 4,263 | 0.46 | - | *LINC00871* | Proband, father-proband |
| chr16:68185354–68204527 | DEL | 1,517 | 0.55 | *NFATC3, AC130462.1, AC020978.6, AC020978.8* | *RPS12P27* | PD-aunt |
| chr16:72321271–72350532 | DEL | 15,501 | 0.56 | *LINC01572* | *U6* | PD-aunt |
| chr19:4297903–4299484 | INS | 5,098 | 0.59 | *TMIGD2* | *FSD1* | PD-aunt |
| chr21:32251481–32266664 | DEL | 5,520 | 0.57 | *AP000265.1* | *MIS18A* | Brother, father-proband, PD-aunt |
| chrX:41053993–41066578 | INS | 3,150 | 0.51 | - | *CLIC4P3* | PD-aunt |

1Genes in bold indicate neuromuscular-related gene candidates. 2Not all entries represent official HUGO gene symbols. Some loci correspond to ENSEMBL-predicted transcripts or gene models that require experimental confirmation.

DEL, deletion; DM1, myotonic dystrophy type 1; INS, insertion; NA, non-applicable; OGM, optical genome mapping; PD, Parkinson’s disease; SV, structural variant; VAF, variant allele frequency.
